# Unidirectional Perpendicularly Aligned Lamella-Structured Oligosaccharide (A) ABA Triblock Elastomer (B) Thin Films Utilizing Triazolium^+^/TFSI^–^ Ionic Nanochannels

**DOI:** 10.1021/acsmacrolett.1c00712

**Published:** 2022-01-03

**Authors:** Johanna Majoinen, Cécile Bouilhac, Patrice Rannou, Redouane Borsali

**Affiliations:** † Université Grenoble Alpes, CNRS, CERMAV, 38000 Grenoble, France; ‡ Université Grenoble Alpes, Université Savoie Mont Blanc, CNRS, Grenoble INP, LEPMI, 38000 Grenoble, France; § Université Grenoble Alpes, CNRS, CEA, INAC-SyMMES, 38000 Grenoble, France; ∥ ICGM, Université Montpellier, CNRS, ENSCM, 34095 Montpellier, France

## Abstract

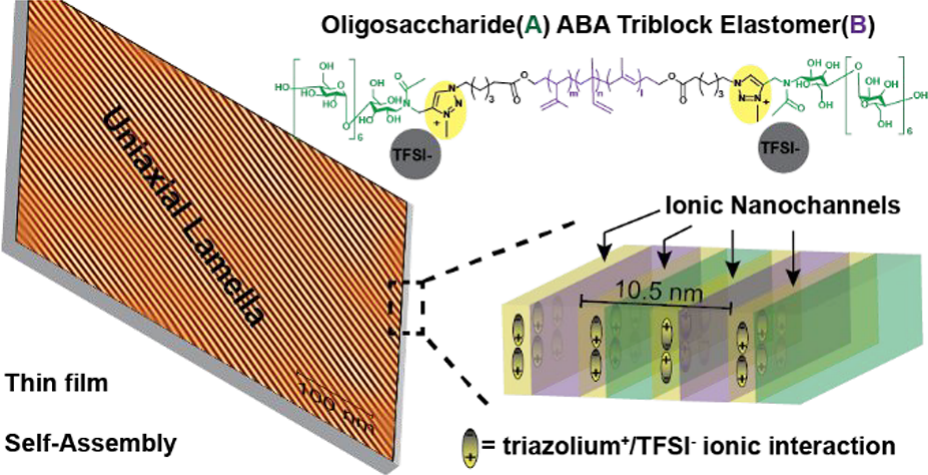

We designed and synthesized high χ-low *N*-maltoheptaose-(triazolium^+^/N(SO_2_CF_3_)_2_^–^)-polyisoprene-(triazolium^+^/N(SO_2_CF_3_)_2_^–^)-maltoheptaose ABA triblock elastomers featuring triazolium^+^/N(SO_2_CF_3_)_2_^–^ (TFSI^–^) counteranion ionic interfaces separating their constituting polymeric sub-blocks. Spin-coated and solvent-vapor-annealed (SVA) MH_1.2k_-(T^+^/TFSI^–^)-PI_4.3k_-(T^+^/TFSI^–^)-MH_1.2k_ thin films demonstrate interface-induced charge cohesion through ca. 1 nm “thick” ionic nanochannels which facilitate the self-assembly of a perpendicularly aligned lamellar structure. Atomic force microscopy (AFM) and (grazing-incidence) small-angle X-ray scattering ((GI)SAXS) characterizations of MH_1.2k_-(T^+^/TFSI^–^)-PI_4.3k_-(T^+^/TFSI^*–*^)-MH_1.2k_ and pristine triBCP analogous thin films revealed sub-10 nm block copolymer (BCP) self-assembly and unidirectionally aligned nanostructures developed over several μm^2^ areas. Solvated TFSI^–^ counterions enhance the oligosaccharide sub-block packing during SVA. The overall BCP phase behavior was mapped through SAXS characterizations comparing di- vs triblock polymeric architectures, a middle PI sub-block with two different molecular masses, and TFSI^–^ or I^–^ counteranion effects. This work highlights the benefits of inducing single-point electrostatic interactions within chemical structures of block copolymers to master the long-range self-assembly of prescribed morphologies.

Synthetic block copolymers (BCPs) are key enabling building blocks toward functional organic materials with *tunable-by-design* properties.^1^ Hierarchical self-assembly of tailored-made macromolecular architectures into prescribed morphologies (featuring cylinders, lamellae, spheres, etc.)^2^ with desired functions becomes experimentally facile when fine-tuning the polymer–polymer interactions (Flory–Huggins parameter, χ) for highly immiscible blocks and their volume fractions (*f*) with an adjusted number of repeating units (*N*). Traditional coil–coil BCP nanostructures with a domain spacing of tens of nanometers (nm) have paved the way toward the so-called high χ-low *N* BCPs to obtain smaller features, down to the sub-10 nm dimensions.^3^ Within high χ-low *N* BCPs, the sub-blocks have high immiscibility^4^ and small *N* values resulting in nanostructures featuring domain sizes as small as 3 nm, highly seeked for nanopatterning applications.^5,6^ Oligosaccharides function simultaneously as sustainable and key-enabling high χ building blocks to allow for sub-10 nm BCP domain sizes.^7−11^ Moreover, oligosaccharide-based BCPs can be elegantly synthesized with ready-made synthetic building blocks utilizing the copper(I)-catalyzed alkyne–azide cycloaddition (CuAAC) click reaction.^12,13^

We designed and synthesized high χ-low *N* maltoheptaose (MH) and polyisoprene (PI) block coelastomers using CuAAC coupling to target triazole junction point functionality.^14^ The MH vs PI sub-block’s high polarity difference leads to high immiscibility and hence high χ.^15^ The flexible PI sub-block consists of an ideal complementary block for BCP processing through providing a soft matrix. In addition to the influence of χ and *f*, supramolecular interactions (e.g., π–π stacking,^16^ hydrogen bonding,^17^ and ionic interactions^18,19^) at the BCP interface have recently demonstrated compelling evidence for enhanced self-assembly, ionic interactions notably inducing unique counterion effects.^19^ Ionically conducting BCP thin films with controlled structure orientation (perpendicular or parallel) with respect to electrodes are highly seeked in order to allow for efficient anisotropic ionic transport.^20^ To date, BCP domain orientation in thin films relies on mastering the subtle interplay of varying energies at polymer–air, polymer–polymer, and polymer–substrate interfaces.^21^ To address this issue, we report on the effect of electrostatic interactions at charge-modified BCP interfaces to control the self-assembly of oligosaccharide-based high χ-low *N* BCPs. MH_1.2k_-(triazolium^+^/TFSI^–^)-PI_4.3k_-(triazolium^+^/TFSI^–^)-MH_1.2k_ triBCP thin films were prepared accordingly, wherein the formation of ionic nanochannels guides the BCP structure orientation (Scheme 1). Generally, controlling the orientation of BCP structures from several μm^2^ up to the cm^2^ range is realized with the help of shearing^22^ and external fields^23^ (e.g., electric or magnetic) or through templating.^24^ Here, we show how charge cohesion of the BCP interfaces promotes unidirectional perpendicular lamella (Lam) nanostructure formation over areas of several μm^2^ for solvent-vapor-annealed^25,26^ (SVA) BCP thin films. We provide a direct vs reciprocal space quantification of sub-10 nm Lam domain spacing (*d*) in thin films with atomic force microscopy (AFM) imaging and grazing-incidence small-angle X-ray scattering (GISAXS), respectively.

**Scheme 1:**
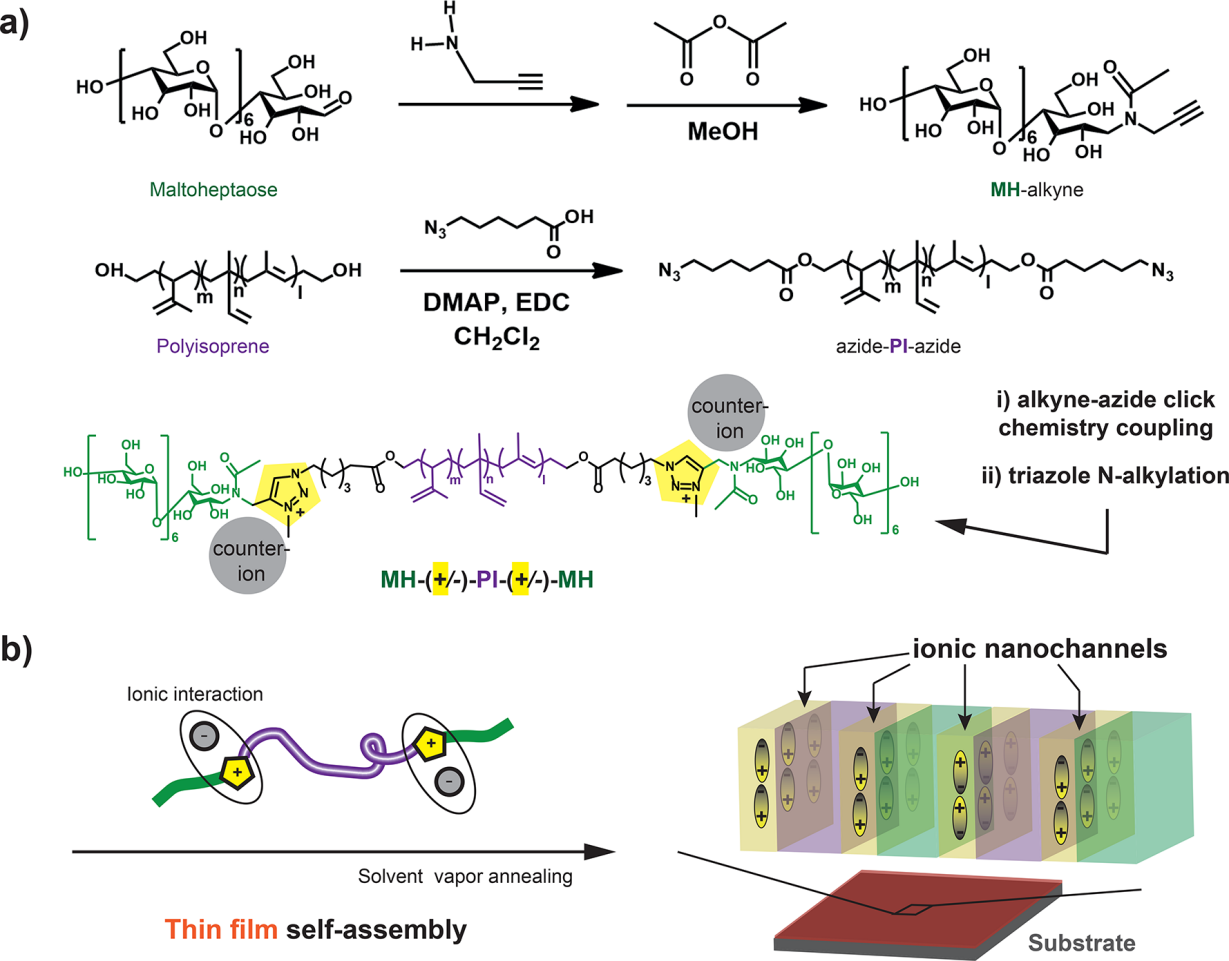
Synthesis (a) of a Charge-Modified ABA Maltoheptaose-(triazolium^+^/counteranion^*-*^)-Polyisoprene-(triazolium^+^/counteranion^*-*^)-Maltoheptaose triBCP with End-Functionalized Maltoheptaose and Polyisoprene Building Blocks Using CuAAC Click Chemistry Coupling and Subsequent *n*-Alkylation of Triazole (T) Junction Units and (b) Schematics for Charge-Modified triBCP Interfaces in Self-Assembled Thin Films with a Perpendicular Lamella-Based Nanostructure and Ionic Nanochannels

Ready-made MH (highly discrete number-average molar mass *M*_n_ distribution, with *M*_n_= 1.2 kg mol^–1^: Figure S1) and hemitelechelic α-monohydroxy-end-capped (*M*_n_= 3.6 kg mol^–1^) and homotelechelic α,ω-bishydroxy-end-capped PIs (*M*_n_ = 4.3 kg mol^–1^ and *M*_n_= 9.0 kg mol^–1^, respectively) were end-functionalized and further used in the synthesis of AB diBCPs and ABA triBCPs through CuAAC (Scheme 1). Coupling reaction conditions and polymer characterizations are described in the Supporting Information (SI) (Figures S2–S8). The reducing chain end of MH was selectively functionalized with an alkyne moiety, using propargyl amine.^27^ PIs (*D* = *M*_w_/*M*_n_ = 1.09–1.25) were modified with 6-azido-hexanoic acid to afford end-functionalized elastomer sub-blocks with an azide function. CuAAC yielded MH_1.2k_-(triazole)-PI_3.6k_ diBCP and MH_1.2k_-(triazole)-PI_4.3k/9k_-(triazole)-MH_1.2k_ triBCP model compounds with 1,2,3-triazole (T) junction units (Table S1 for polymer properties). Finally, a straightforward route to the BCP ionic interface is demonstrated by *n*-alkylation of triazole ring(s) with *N*-methyl bis[(trifluoromethyl)sulfonyl]imide (MeTFSI) or iodomethane (MeI), creating a methyltriazolium (T^+^)/counteranion^–^ (TFSI^–^ or I^–^) junction separating the sub-blocks. Full conversion of T junctions to T^+^/TFSI^*–*^ using MeTFSI was verified with ^1^H NMR spectroscopy (Figures S9 and S10). N-Alkylation with MeI did not reach full conversion, even with prolonged reaction times, ambient temperature, or an extra amount of reagent (Figures S9 and S11). The T^+^/I^*–*^ interface is thermally unstable, resulting in N-demethylation.^28^ Moreover, MeI reacts with PI double bonds by coordination bonding, even covalently,^29^ reducing the triazole-to-methyltriazolium (T-to-T^+^) conversion. The results related to MeI are therefore presented here as a normative base for comparing effects of the counterion (TFSI^–^ or I^–^) onto BCP self-assembly.

BCP glass transition temperatures (*T*_g_) were determined with differential scanning calorimetry (DSC) (Figures S12–14 and Table S1). *T*_g_ values varied in between 5 and 19 °C for pristine and charge-modified BCPs. PI backbone structures rich in 1,2- and 3,4-addition typically have higher *T*_g_ values than PIs exhibiting only 1,4-structure.^30^ Thermal transitions observed during the first heating scan could not be reproduced during the second cycle for any of the BCPs studied. Order–disorder transition temperature (*T*_ODT_) for MH-based BCPs with high χ is expected to be high, possibly unobtainable before triggering the BCP thermal degradation. Further annealing steps during DSC measurements below BCP *T*_ODT_ were not performed in order to avoid their chemical degradations.^31^

Variable-temperature SAXS characterizations for all di- and triBCPs were performed to quantify the effect of thermal treatment on nanostructure formation for the investigated polymer architectures (Figure 1). We discuss the effect of the counteranion on phase behavior and, additionally for pristine vs charge-modified triBCPs, the effect of two different *M*_n_ values for the PI middle sub-block.

**Figure 1:**
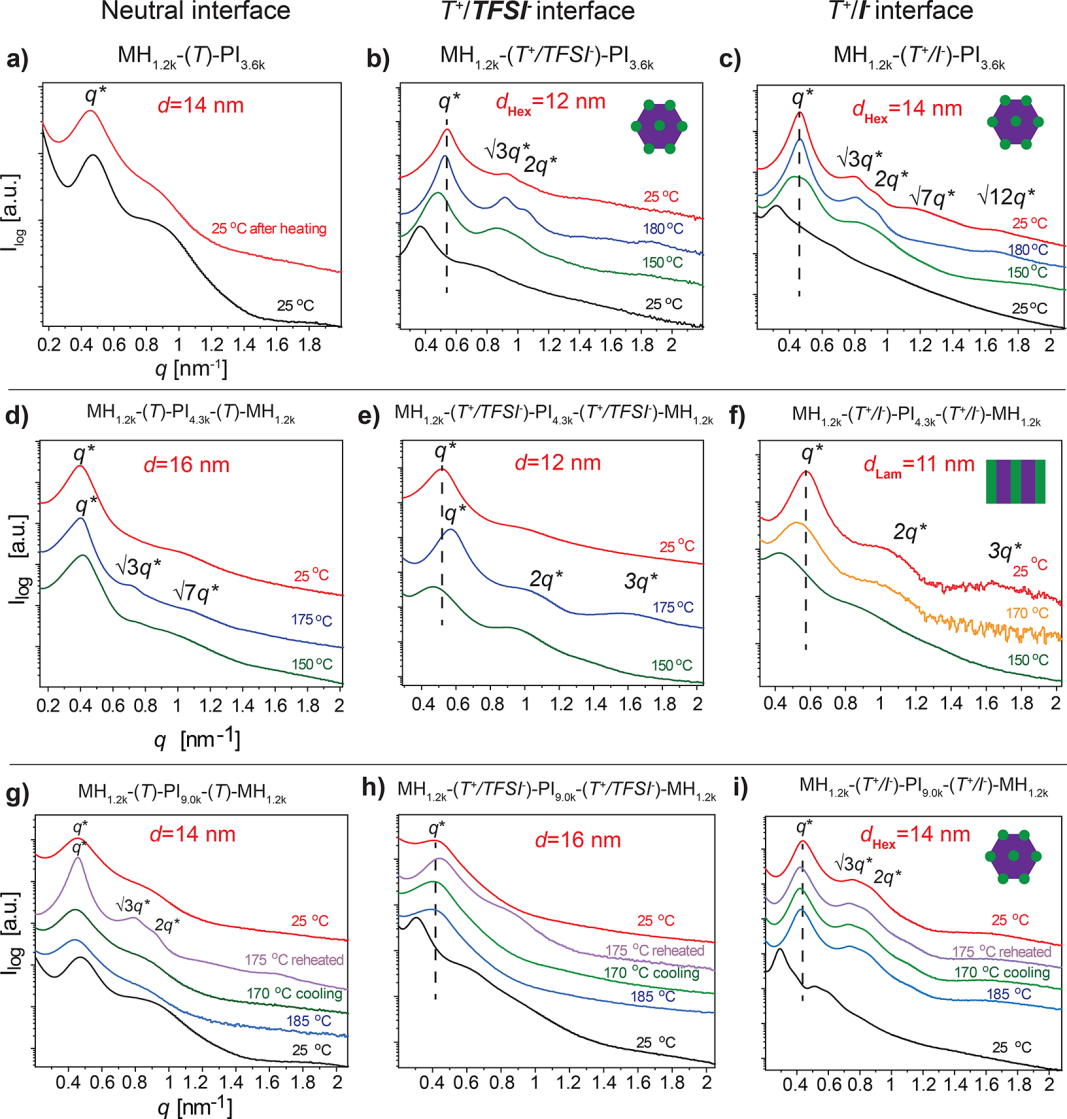
Selected data (including *d* = 2π/*q* values) from variable-temperature SAXS characterizations (heating scan from 25 to 185 °C followed by a cooling scan down to 25 °C) of pristine (neutral *T* unit) (a) and charge-modified (T^+^/TFSI^*–*^ or T^+^/I^*–*^ junction units) (b,c) diBCPs, (d–f) triBCPs with 4.3 kg mol^–1^ for the PI middle block, and (g–i) triBCPs with 9.0 kg mol^–1^ for the PI middle block.

The downsizing effect of thermal treatment onto the domain spacing (*d*) for charge-modified BCPs is evident from the comparison of the primary scattering peak (q*) positions after heating (Figure 1b, c, e, f, h, i; dashed vertical lines). Pristine di- and triBCP q* positions remain unchanged and with a broad profile (Figure 1a, d, g). A clear enhancement for the hexagonally packed cylinder (Hex) morphology is observed in SAXS profiles for MH_1.2k_-(T^+^/TFSI*^–^*)-PI_3.6k_ and MH_1.2k_-(T^+^/I^*–*^*)*-PI_3.6k_ diBCPs with Bragg reflections (Figure 1b,c) displaying *q*/q* ratios: 1, √3, 2, √7, and √12. MH cylinders in the PI matrix are indicated with *f*_PI_ = 86.^15^ MH_1.2k_-(T^+^/TFSI^*–*^)-PI_3.6k_ with the T^+^/TFSI^–^ junction has a reduced *d* = 12 nm compared to the pristine diBCP (*d* = 14 nm) (Figure 1a,b). The Hex phase evolves between 150 and 180 °C for both charge-modified diBCPs. Interestingly, MH_1.2k_-(T^+^/I^*–*^)-PI_3.6k_ and pristine diBCP Hex morphologies present the same *d* values. *d* remains unaffected possibly because of partial T-to-T^+^/I^–^ conversion, leading to incomplete ion connectivity between the interfaces.

The charge-modified MH_1.2k_-(T^+^/TFSI^*–*^)-PI_4.3k_-(T^+^/TFSI^*–*^)-MH_1.2k_ and MH_1.2k_-(T^+^/I^*–*^)-PI_4.3k_-(T^+^/I^*–*^)-MH_1.2k_ triBCPs both show the Lam morphology at 170 °C, with a considerable decrease of *d* of ca. 5 nm when compared to pristine MH_1.2k_-(T)-PI_4.3k_-(T)-MH_1.2k_ with *d* = 16 nm (Figure 1d–f). The MH_1.2k_-(T^+^/I^*–*^)-PI_4.3k_-(T^+^/I^*–*^)-MH_1.2k_ analogue preserves the long-range order with decreased *d* after heating. Compared to diBCPs, the ABA triBCP architecture with polydisperse middle B block can experience packing frustrations during phase transition.^32^ Exemplifying this phenomenon, pristine triBCP with a dispersed (*D* = 1.25) PI_4.3k_ middle block and *f*_PI_ = 78 resulted in a Hex phase with a curved interface. Counterion mobility and solubility can either suppress or enhance BCP nanostructure formation,^33^ with confined counterions at the interface possibly suppressing phase separation as a consequence of an entropic penalty. TriBCPs with the PI_9.0k_ middle block (*D* = 1.09) and *f*_PI_ = 88 form Hex phases regardless of the counterion, yet with a smaller *d* for MH_1.2k_-(T)-PI_9k_-(T)-MH_1.2k_ compared to the T^+^/TFSI^–^ interface triBCP supporting the effects of middle-block dispersity on phase separation (Figure 1d,g). MH_1.2k_-(T^+^/I*^–^*)-PI_3.6k_, MH_1.2k_-(T^+^/I^*–*^)-PI_4.3k/9.0k_-(T^+^/I^*–*^)-MH_1.2k_ diBCP, and triBCP SAXS data illustrate that T-to-T^+^/I^*-*^ full conversion is needed neither to achieve long-range order (Figure 1c, f, i) nor to decrease the *d*. The bulky TFSI^–^ counterion can disrupt block–block interactions restraining crystallization for rod–coil BCPs.^19^ Similar disturbed interactions are evidenced in MH_1.2k_-(T^+^/TFSI^*–*^)-PI_4.3k_-(T^+^/TFSI^*–*^)-MH_1.2k_ and MH_1.2k_-(T^+^/TFSI^*–*^)-PI_9k_-(T^+^/TFSI^*–*^)-MH_1.2k_ SAXS data, as MH hydrogen bonding and T^+^/TFSI^–^ interface interactions hamper the phase transition (Figure 1e,h). Pristine MH_1.2k_-(T)-PI_9k_-(T)-MH_1.2k_ higher-order reflections appeared on reheating to 185 °C, but the primary scattering peak (q*) remained broad and of low intensity after heating, indicating a rather disordered final nanostructure (Figure 1g).

As the effect of the T^+^/TFSI^–^ interface on enhancing the triBCP bulk self-assembly seemed moderate according to variable-temperature SAXS, we next focused our attention on thin film self-assembly and solvent vapor annealing (SVA). AFM was used to image MH_1.2k_-(T^+^/TFSI^*–*^)-PI_4.3k_-(T^+^/TFSI*^–^*)-MH_1.2k_ and MH_1.2k_-(T)-PI_4.3k_-(T)-MH_1.2k_ triBCP thin films prepared by spin-coating on plasma-treated silicon wafers (see details in the SI). To assess the effect of the T^+^/TFSI^*–*^ interface on thin film morphology and structure orientation related to the substrate surface, the BCP film thickness, substate surface energy, and SVA time with solvent composition used were kept constant throughout the experiments. SVA was performed in a closed container for 24 h using a THF/H_2_O 90:10 wt % solvent mixture placed next to the thin film. A film thickness of ca. 21 nm after SVA was determined with AFM from film scratch height. As-cast MH_1.2k_-(T^+^/TFSI^*–*^)-PI_4.3k_-(T^+^/TFSI*^–^*)-MH_1.2k_ and MH_1.2k_-(T)-PI_4.3k_-(T)-MH_1.2k_ thin films displayed an initial disordered surface topography (Figure S18). After SVA, the differences in the film topographical features became highly salient (Figure 2). As the pristine MH_1.2k_-(T)-PI_4.3k_-(T)-MH_1.2k_ thin film fingerprint lines align locally, forming small-size granular domains (highlighted in Figure 2c,d with circles), the charge-modified MH_1.2k_-(T^+^/TFSI^*–*^)-PI_4.3k_-(T^+^/TFSI^*–*^)-MH_1.2k_ thin film features unidirectionally aligned lines across the image (Figures 2a,b and S16), over distances of 500 nm. The line spacing (*L*) extracted from FFTs indicated a *d* value of 11 nm (*L* = 5.5 nm) for MH_1.2k_-(T^+^/TFSI^*–*^)-PI_4.3k_-(T^+^/TFSI^–^)-MH_1.2k_ and *d* value of ca. 14 nm for MH_1.2k_-(T)-PI_4.3k_-(T)-MH_1.2k_ (*L* varies between 6.6 and 7.4 nm) (Figure 2a,c). The MH_1.2k_-(T^+^/TFSI^*–*^)-PI_4.3k_-(T^+^/TFSI^*–*^)-MH_1.2k_ thin film lines appear hazy (Figure 2a). 3D visualization of a MH_1.2k_-(T^+^/TFSI^–^)-PI_4.3k_-(T^+^/TFSI*^–^*)-MH_1.2k_ thin film height image shows a surface roughness of ca. 4 nm, as the higher surface energy PI block protrudes at the air–polymer surface (Figure S15). Nanostructure orientations are highlighted with 2D FFT filtering (Gwyddion software) (Figure 2b,d). Filtered separated data (difference image in Figure 2b inset) reveal clear bright/dark alternating lines. Applying the same filtering procedure for areas in the ellipsoidal FFT profile of the MH_1.2k_-(T)-PI_4.3k_-(T)-MH_1.2k_ thin film allows distinguishing the local granular domain alignment (red and blue circles in Figure 2d) with varying line orientations (red and blue arrows Figure 2d).

**Figure 2:**
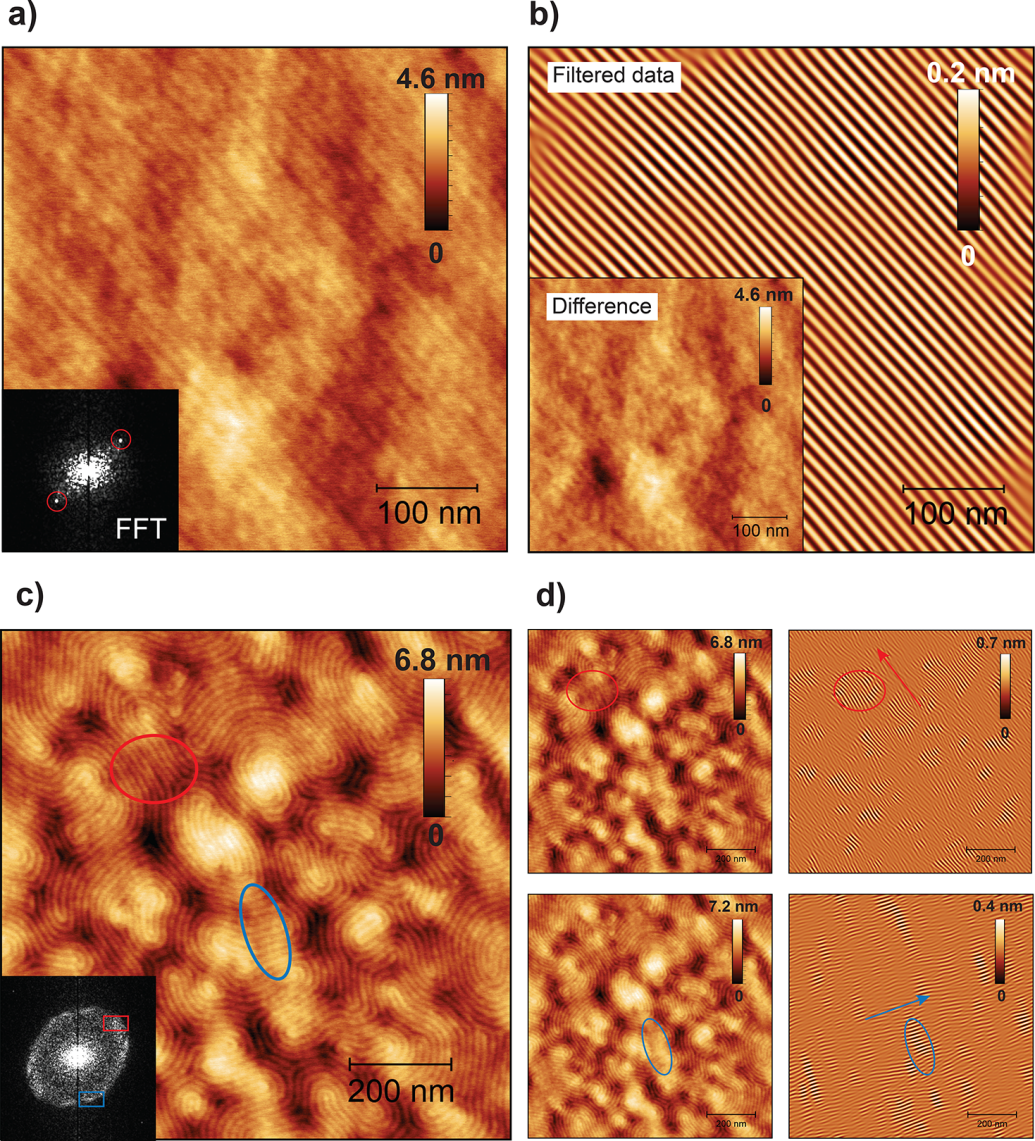
AFM height images for (a) charge-modified MH_1.2k_-(T^+^/TFSI*^–^*)-PI_4.3k_-(T^+^/TFSI^*–*^)-MH_1.2k_ versus (c) pristine MH_1.2k_-(T)-PI_4.3k_-(T)-MH_1.2k_ triBCP thin films. Fast Fourier transforms (FFTs) associated with an AFM topography image are used in image filtering in (b) and (d) presented with a *difference* image of filtered data and a *filtered* data image to clarify the unidirectional vs local line orientation between the two films.

Figure 3 further confirmed that the MH_1.2k_-(T^+^/TFSI^*–*^)-PI_4.3k_-(T^+^/TFSI*^–^*)-MH_1.2k_ thin film unidirectional line orientation extends up to the 5 μm^2^ area. Curiously, careful AFM imaging revealed minority areas with hexagonal arrays of isolated dots on the surface complicating the structure interpretation (Figures 3, image 4, and S17).

**Figure 3:**
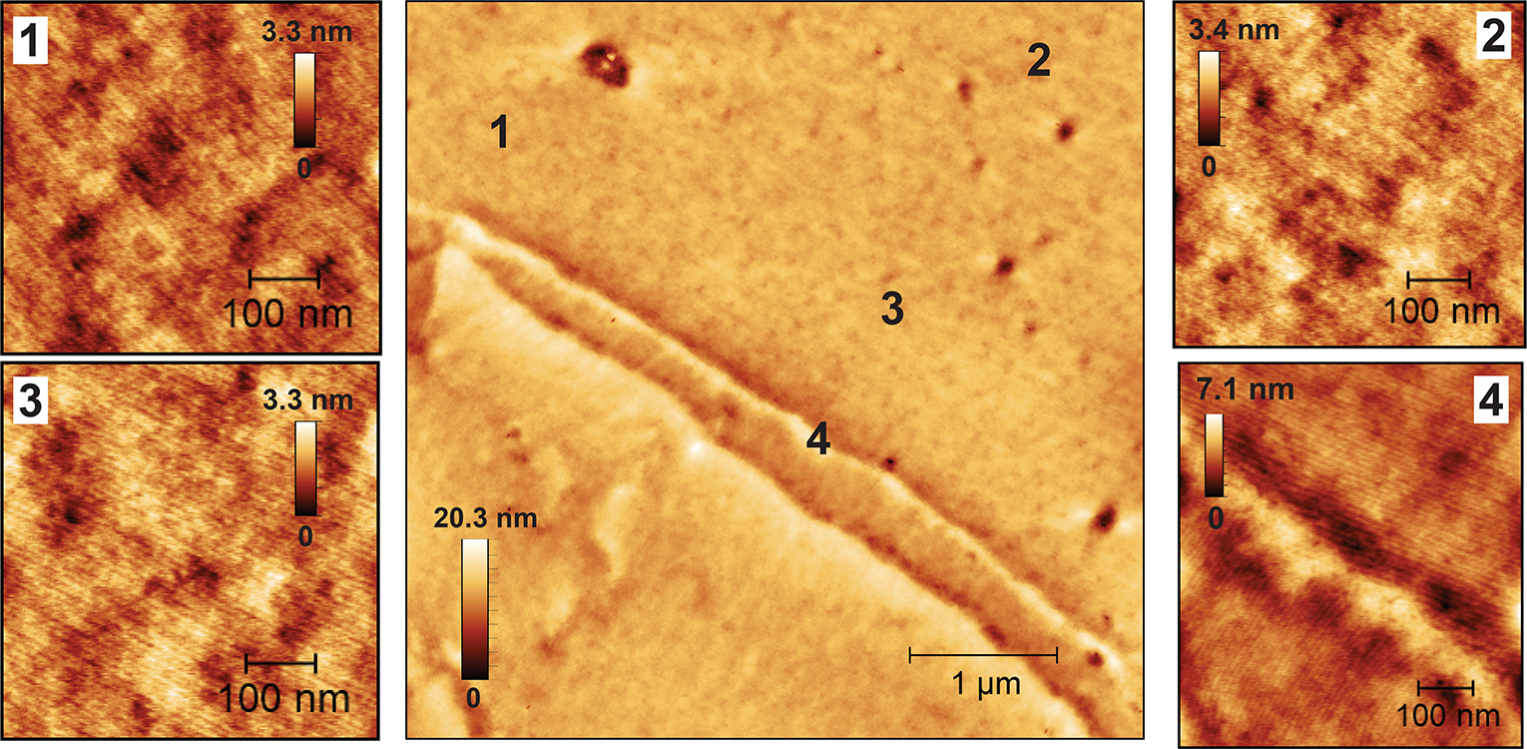
AFM height analyses of MH_1.2k_-(T^+^/TFSI^*–*^)-PI_4.3k_-(T^+^/TFSI^*–*^)-MH_1.2k_ thin films (5 μm^2^ area). Zoom-in images numbered 1–4. Image 4 shows a defect edge, where uniaxial lines appear perpendicular to each other.

GISAXS characterizations confirmed the morphologies for MH_1.2k_-(T^+^/TFSI^*–*^)-PI_4.3k_-(T^+^/TFSI^*–*^)-MH_1.2k_ and MH_1.2k_-(T)-PI_4.3k_-(T)-MH_1.2k_ thin films (Figure 4). Charge-modified triBCP showed a clear in-plane Lam scattering pattern (q* = 0.6, 2q* = 1.2) with a *d*_charged_ value of 10.5 nm (Figure 4a,b). Whereas sharp spots are clearly seen at the in-plane Yoneda line for the T^+^/TFSI^*–*^ interface (Figure 4a,b), a pristine MH_1.2k_-(T)-PI_4.3k_-(T)-MH_1.2k_ thin film displays broad peaks with Debye–Scherrer rings (pointed with arrows in Figure 4d,e) attributed to the tilted domain orientations within the thin film.^34^ A Lam nanostructure is also obtained for the MH_1.2k_-(T)-PI_4.3k_-(T)-MH_1.2k_ thin film (q* = 0.48, 2q* = 0.95) with *d*_neutral_ = 13 nm (Figure 4e) in full agreement with AFM analyses. Intense rod-shaped out-of-plane reflections are seen for both pristine and charge-modified thin films (Figure 4a and c and 4d and f). These Bragg rods result from incident beam reflection from an ultrathin BCP film where the perpendicular Lam nanostructure is aligned parallel with respect to the reflected beam^35^ (schematics Figure 4a). The charge-modified triBCP thin film produces a sharp and narrow Bragg rod compared to the broad and somewhat distorted one shown by the pristine triBCP thin film due to Debye–Scherrer rings distorting the rod reflections (Figure 4a,c and 4d,f). GISAXS data for as-cast MH_1.2k_-(T^+^/TFSI*^–^*)-PI_4.3k_-(T^+^/TFSI^*–*^)-MH_1.2k_ and MH_1.2k_-(T)-PI_4.3k_-(T)-MH_1.2k_ thin films did not show notable structure development. (Figure S18). Evidence for the Hex phase formation in thin films was not observed with GISAXS. We therefore conclude on Lam structure formation for MH_1.2k_-(T^+^/TFSI^*–*^)*-*PI_4.3k_-(T^+^/TFSI^*–*^)*-*MH_1.2k_, both in bulk and for the thin film, regardless of *f*_PI_ = 78 (Figure 1e and 1a–c). The AFM imaging of a grain boundary with merged facing line frontiers (Figure S16) supports the claims that dotted features (Figures 3 image 4 and S17) are nonequilibrium structures within a swollen BCP thin film. This is also compatible with dispersity-induced PI_4.3k_ middle-block packing frustrations which affect the evolution of the surface morphology.^32^

**Figure 4:**
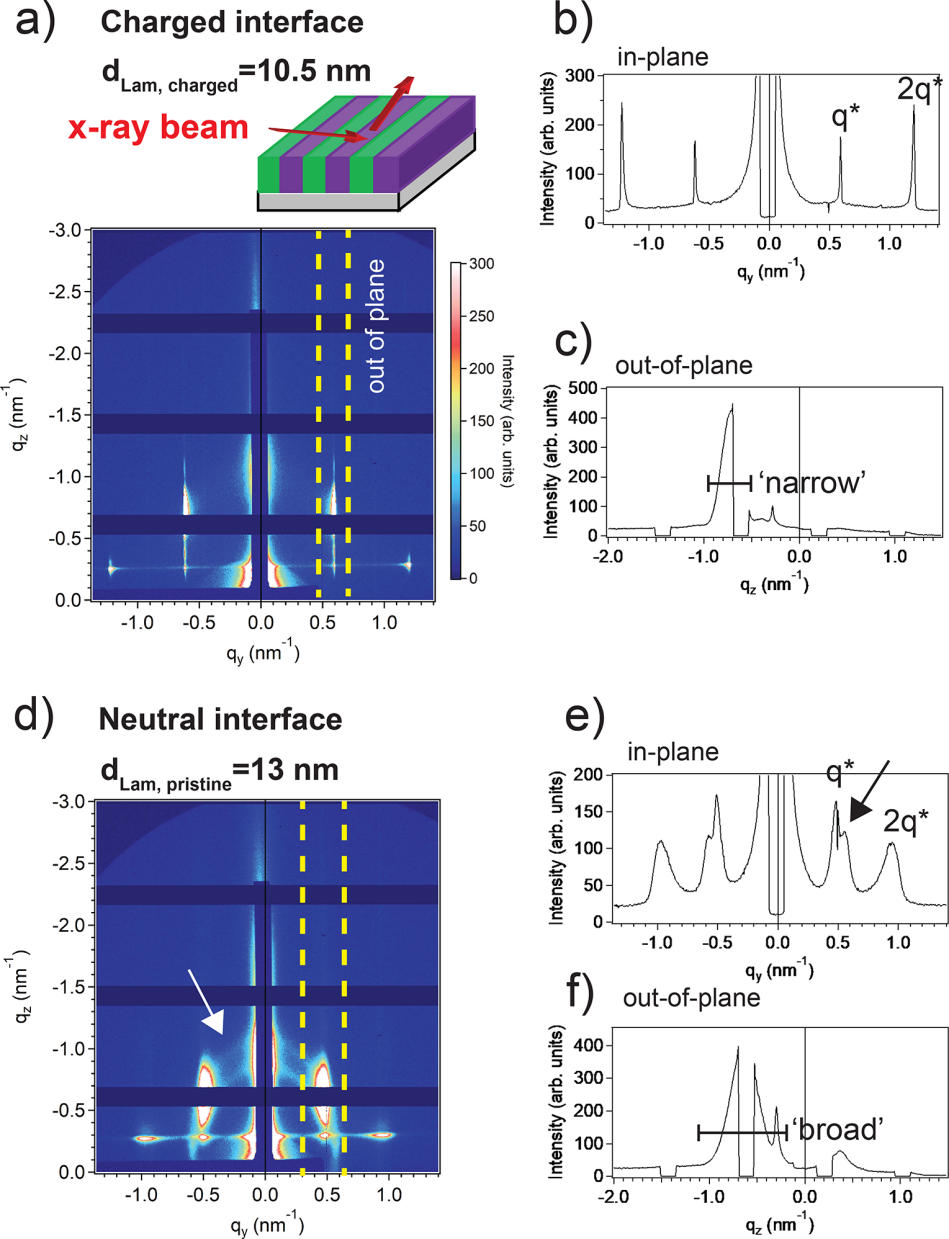
GISAXS results: (a) 2D SAXS pattern recorded for a MH_1.2k_-(T^+^/TFSI^*–*^)-PI_4.3k_-(T^+^/TFSI^*–*^)-MH_1.2k_ thin film combined with a scheme showing the X-ray beam configuration with respect to the Lam nanostructure. (b,c) In-plane vs out-of-plane corresponding 1D scattering profiles. (d) 2D SAXS pattern acquired onto a MH_1.2k_-(T)-PI_4.3k_-(T)-MH_1.2k_ thin film. Note that out-of-plane regions are indicated with yellow dashed lines and Debye–Scherrer rings highlighted with a white arrow. (e,f) In-plane vs out-of-plane corresponding 1D scattering profiles.

Oligosaccharide-based BCPs are highly responsive to SVA^7^ and microwave annealing.^36^ Modeling studies have revealed that the solvation of the TFSI^–^ counteranions with water molecules is imparted by strong hydrogen-bonding interactions^37^ and TFSI^–^ anions contributing to the nanophase segregation process at play within thin films of MH-based triBCPs. Tetrahydrofuran promotes the mobility of the PI sub-block during SVA, while minute amounts of water facilitate interactions of TFSI^–^ with MH sub-blocks. Importantly, the THF/water mixture offers a neutral top layer for both MH and PI sub-blocks to emerge at the polymer-THF/water interface, promoting an equilibrium Lam formation, possibly transiting through a perpendicular Hex top layer structure before stabilizing into a final perpendicularly aligned Lam^38^ (Figure 3 and S17).

The MH_1.2k_-(T^+^/TFSI^*–*^)-PI_4.3k_-(T^+^/TFSI*^-^*)-MH_1.2k_ Lam nanostructure depends on the ca. 21 nm thin film thickness (*t*), which is commensurate with the *d*_charged_ = 10.5 nm of the Lam (*t*/*d*_charged_ = 2). We suggest that strong electrostatic interactions of T^+^/TFSI^–^ trigger the self-assembly of well-connected channels, enabling the unidirectional Lam alignment with a 2.5 nm decrease in *d*, regardless of the high *f*_PI_ (Figure 5). Luo et al. reported on locally aligned structures for thermally annealed and reactive ion-etched PDMS_1.7k_-*b*-PMMA_5.1k_ thin films, unaffected by the presence of an ionic junction interfacing the PDMS and PMMA sub-blocks.^18^ Vice versa, Ji et al. reported on poly(3-hexylthiophene) amorphization due to a T^+^/TFSI^–^ interface hampering with rod-block π–π interactions upon thermal annealing of poly(3-hexylthiophene)-*b*-poly(methyl methacrylate).^19^ As we rely on a thin film thickness commensurate with the domain spacing (*vide supra*) and SVA for enhancing TFSI^–^ mobility, the electrostatic interactions prevail, leading to well-connected T^+^/TFSI^–^ interfacial ionic nanochannels. To the best of our knowledge, this is the first report of a long-range unidirectional alignment for a BCP Lam nanostructure induced by electrostatic interactions within ionic nanochannels defined by T^+^*/*counteranion^–^ junctions separating polymeric sub-blocks. We emphasize on utilizing SVA with optimized BCP film thicknesses to enable strong electrostatic interactions within ionic nanochannels to result in long-range aligned microphase-separated morphologies. Increasing the *f*_PI_*M*_n_ to 9.0 kg mol^–1^ in triBCP thin film indicates the formation of a Hex phase (Figure 1g,h,i) and requires rebalancing the surface energies for the successful formation of ionic nanochannels.

**Figure 5:**
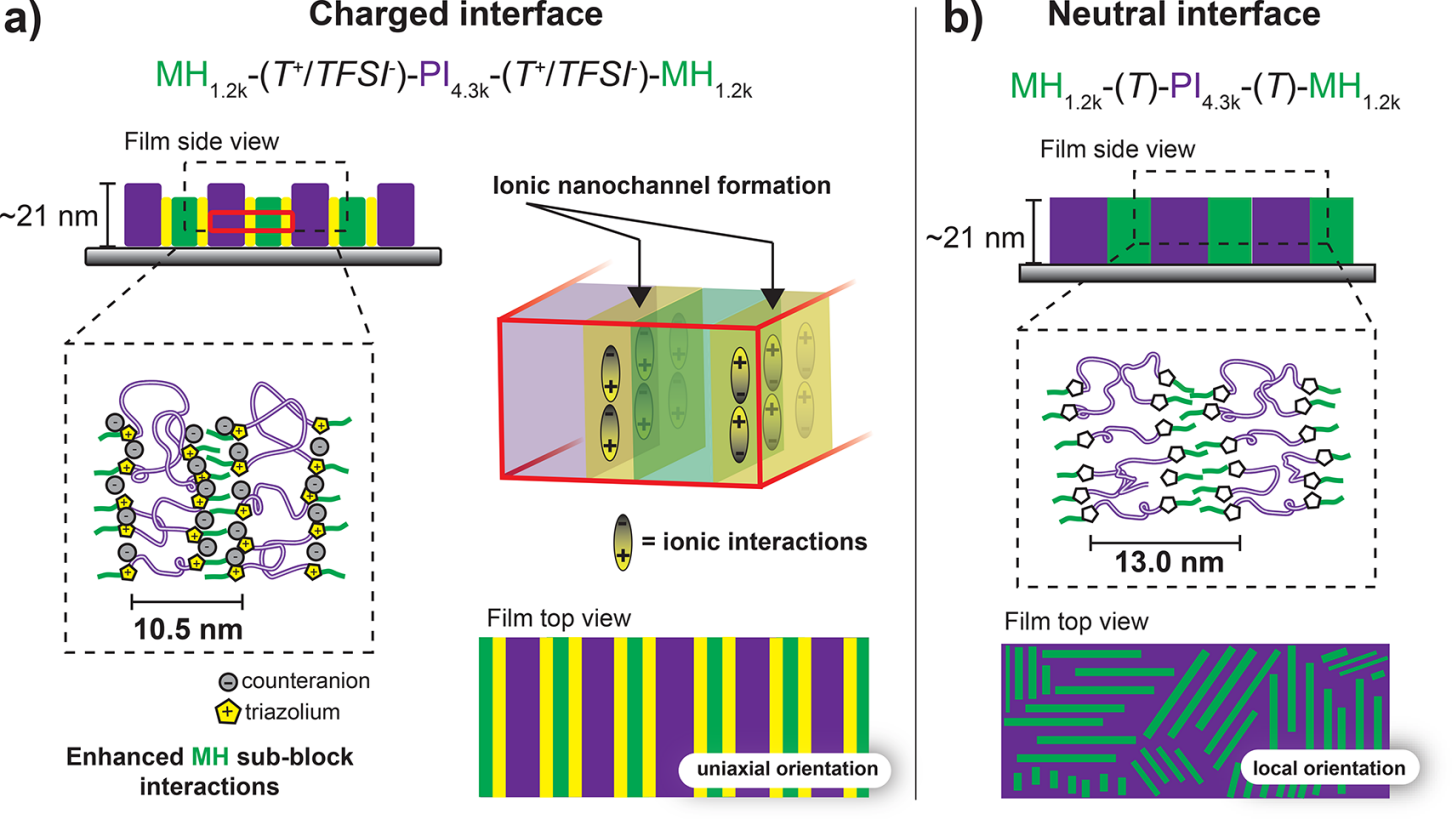
Suggested effects of (a) charged interface (T^*+*^/TFSI^–^)*-*induced charge cohesion for the self-assembly of MH_1.2k_-(T^+^/TFSI^–^)-PI_4.3k_-(T^+^/TFSI^–^)-MH_1.2k_ triBCP thin films and of (b) a neutral (T) interface for the self-assembly of MH_1.2k_-(T)-PI_4.3k_-(T)-MH_1.2k_ triBCP thin films. Charged T^+^/TFSI^–^ junction units self-assembled into ionic nanochannels promoting stong interactions among MH sub-blocks, while T interfaces are less effective, leading to irregular interactions between MH sub-blocks. The ionic connectivity is schematically illustrated with triazolium (T^+^) rings in yellow versus TFSI^–^ counteranions in gray.

To summarize, we show how one can leverage copper(I)-catalyzed alkyne–azide cycloaddition (CuAAC) click chemistry and *n*-alkylated triazolium^+^/counteranion^–^ junctions to enhance the bulk and thin film self-assembly of high χ-low *N* diblock and triblock copolymers using maltoheptaose (MH) and polyisoprene (PI) sub-blocks. Full T-to-T^+^/TFSI^–^ conversion is obtained using MeTFSI in n-alkylation. Interestingly, despite conversion as low as 11% for T-to-T^+^/I^–^, SAXS studies confirmed enhanced BCP microphase separation, illustrating the powerfulness of T^+^/counteranion^–^ junctions to fine-tune BCP self-assembly. TFSI^–^ solubilization during SVA of the BCP thin film together with optimized film thickness enable long-range-ordered unidirectional perpendicular Lam formation, quantified for MH_1.2k_-(T^+^/TFSI^–^)-PI_4.3k_-(T^+^/TFSI^–^)-MH_1.2k_ thin film with both real-space (AFM) and reciprocal-space (GISAXS) analyses. Large areas of several μm^2^ featuring sub-10 nm charge-modified triBCP domains are remarkably uniform and easily fabricated. Long-range nanostructure orientation is conveniently achieved without shearing or relying on graphoepitaxy, highlighting how electrostatic interactions facilitate BCP domain alignment through ionic nanochannel formation. Considering the library of BCPs accessible through CuAAC click chemistry of hemitelechelic and homotelechelic synthetic or biosourced/based polymeric building blocks, thereby amenable to n-alkylation of triazole junction units, this study opens doors to explore long-range ordered BCP thin films for advanced nanopatterning (nanoelectronics) or efficient and dimensionality-controlled ionic transport for electrochemical energy storage (nanoionics).

## Supporting Information Available

Individual polymer block analyses (Maldi-ToF mass spectrometry), BCP syntheses and characterizations (^1^H NMR, FT-IR), DSC, additional AFM images, and GISAXS analyses (PDF)
