# Supplementary material for: Unidirectional Perpendicularly Aligned Lamella-Structured Oligosaccharide (A) ABA Triblock Elastomer (B) Thin Films Utilizing Triazolium+/TFSI– Ionic Nanochannels
Source: ACS Macro Lett. 2022 Jan 3;11(1):140–8. doi: 10.1021/acsmacrolett.1c00712 (PMC8772381; doi:10.1021/acsmacrolett.1c00712)
Supplement: Supplementary file 1 — mz1c00712_si_001.pdf [file mz1c00712_si_001.pdf]

Supporting Information (SI) for

Unidirectional Perpendicularly Aligned Lamella-Structured Oligosaccharide (A) ABA Triblock Elastomer (B) Thin Films Utilizing Triazolium$^+$/TFSI$^-$ Ionic Nanochannels

Johanna Majoinen[#,§,†,*] Cécile Bouilhac[&], Patrice Rannou[//,§,*], Redouane Borsali[#,*]

[#]Univ. Grenoble Alpes, CNRS, CERMAV, 38000 Grenoble, France
[//]Univ. Grenoble Alpes, Univ. Savoie Mont Blanc, CNRS, Grenoble INP, LEPMI, 38000 Grenoble, France
[§] Univ. Grenoble Alpes, CNRS, CEA, INAC-SyMMES, 38000 Grenoble, France
[&]ICGM, Univ. Montpellier, CNRS, ENSCM, F-34095 Montpellier, France

[†]Present address: Aalto University, Department of Bioproducts and Biosystems, FI-00076 Espoo, Finland

*Email: johanna.majoinen@aalto.fi,
*Email: patrice.rannou@grenoble-inp.fr
*Email: redouane.borsali@cermav.cnrs.fr

Table of Contents:

- Matrix-Assisted Laser Desorption/Ionization Time-of-Flight Mass Spectrometer (MALDI ToF-MS) analysis on pristine MH
- Table S1 for di/tri-Block CoPolymer (diBCP/triBCP) properties
- diBCP & triBCP syntheses and characterizations
- Triazole *n*-alkylation with N-Methyl bis[(trifluoromethyl)sulfonyl]imide (MeTFSI) and characterization
- Differential Scanning Calorimetry (DSC)
- Variable temperature Small Angle X-ray Scattering (SAXS)
- Thin film preparation
- Atomic Force Microscopy (AFM): Additional images
- Grazing Incidence Small Angle X-ray Scattering (GISAXS)

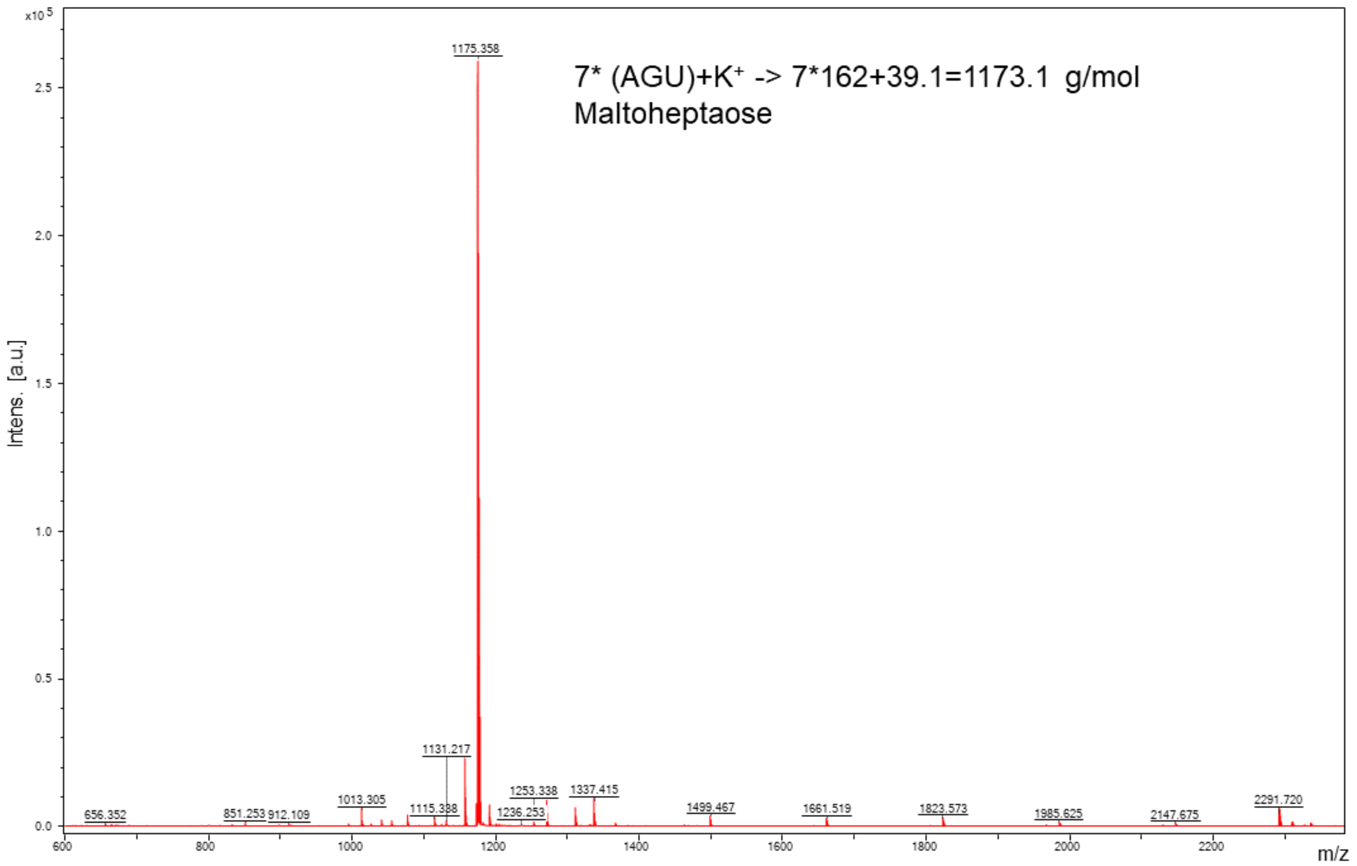

Figure S1. Pristine MaltoHeptaose (MH) oligomer: MALDI-ToF MS spectrum

Table S1. diBCP & triBCP properties

Block copolymer	PI MW[a] [kDa]	PDI_{PI}[b]	f_{PI}[c]	T_g DSC [°C]	$peak_{DSC}$[d] [°C]	Structure & d_{SAXS} after thermal annealing [nm]	Phase transition[e] T_{SAXS} [°C]
$MH_{1.2k}$-(T)-$PI_{3.6k}$	3.6	1.09	86	5	148 exo	*14	-
**$MH_{1.2k}$-(T^+/$TFSI^-$)-$PI_{3.6k}$**	3.6	1.09	86	8	137/192 exo	Hex 12	180
$MH_{1.2k}$-(T^+/I^-)-$PI_{3.6k}$	3.6	1.09	86	5	150/190 exo	Hex 14	180
$MH_{1.2k}$-(T)-$PI_{4.3k}$-(T)-$MH_{1.2k}$	4.3	1.25	78	11	170 exo<br>187 endo	*16	175
**$MH_{1.2k}$-(T^+/$TFSI^-$)-$PI_{4.3k}$-(T^+/$TFSI^-$)-$MH_{1.2k}$**	4.3	1.25	78	19	189 exo	*12	175
$MH_{1.2k}$-(T^+/I^-)-$PI_{4.3k}$-(T^+/I^-)-$MH_{1.2k}$	4.3	1.25	78	11	168 endo	Lam 11	170
$MH_{1.2k}$-(T)-$PI_{9.0k}$-(T)-$MH_{1.2k}$	9.0	1.14	88	6	152/159 exo	*14	150
**$MH_{1.2k}$-(T^+/$TFSI^-$)-$PI_{9.0k}$-(T^+/$TFSI^-$)-$MH_{1.2k}$**	9.0	1.14	88	11	172 exo	*16	170
$MH_{1.2k}$-(T^+/I^-)-$PI_{9.0k}$-(T^+/I^-)-$MH_{1.2k}$	9.0	1.14	88	6	175 exo	Hex 14	175

[a]According to NMR analyses. [b]Determined from SEC analyses. [c]Calculated using N from NMR and $\rho_{maltoheptaose}$=1.85 g/cm^3 and $\rho_{polyisoprene}$=0.92 g/cm^3, [d]Reported values correspond to DSC peak' highest points. *The domain spacing ($d_{SAXS}=2\pi/q$) values have been extracted from the primary peak after thermal annealing of (bulk) materials. [e]Temperature selected from SAXS variable temperature series, where a phase transition could be unambiguously observed.

Di/triblock copolymer syntheses and characterizations

General procedures for synthesis of (maltoheptaose MH)-*b*-polyisoprene PI) di/triBCPs

Materials: *N*-maltoheptaosyl-3-acetamido-1-propyne (MH-C≡CH) was prepared following a reported method.[1] α-monohydroxy-terminated hemitelechelic and α,ω-dihydroxy-terminated homotelechelic Poly(Isoprene)s (PIs: 1,2 and 3,4-addition) ($PI_{3.6k}$-OH and OH-$PI_{4.3k/9.0k}$-OH), with respective number-average molar mass (M_n) of 3.0 kg.mol^{-1}, 3.5 g.mol^{-1}, and 9.0 kg.mol^{-1} and dispersity ($Đ_w$=M_w/M_n) of 1.09, 1.25, and 1.14 (data from supplier), were purchased from Polymer Source, Inc (Montreal, Quebec, Canada). 6-bromohexanoic acid (Aldrich, 97%), sodium azide (NaN_3, Alfa Aesar, 99%), 4-(dimethylamino)pyridine (DMAP, Aldrich, ≥99%), 1-(3-dimethylaminopropyl)-3-ethylcarbodiimide hydrochloride (EDC, TCI Europe, >98%) and copper nanopowder (Cu(core)/CuO(shell), Alfa Aesar, 99.9%) were used as received. Dry dichloromethane (CH_2Cl_2, ≥99.8%, water content ≤0.001%) was purchased from Aldrich and used as received. CupriSorb resin was purchased from Seachem and used as received. Other reagents and solvents were used without further purification.

Before use, the commercial OH-$PI_{4.3k}$-OH was characterized by ^{1}H NMR spectroscopy (**Fig S2**). NMR spectra for all BCPs were recorded using a Bruker Avance DRX 400 MHz.

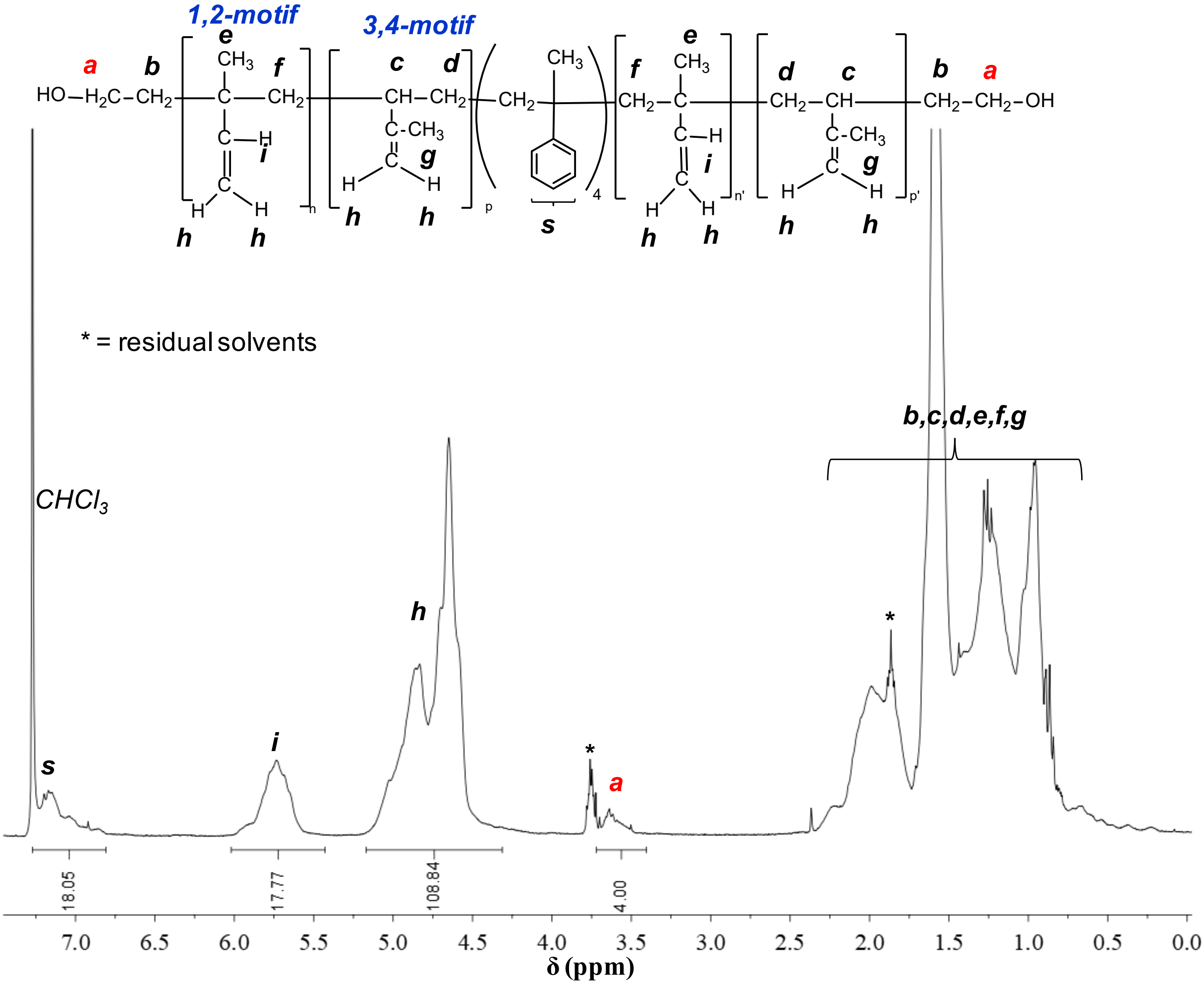

Figure S2. ^{1}H NMR spectrum ($CDCl_3$) of the (commercially available) α,ω-dihydroxy-terminated homotelechelic OH-$PI_{4.3k}$-OH ($M_{n,NMR}$= 4.3 kg.mol^{-1}).
Peak assignment ^{1}H NMR in $CDCl_3$: 7.30-6.79 (broad, C_6H_5- from initiator for PI synthesis, poly(α-methylstyrene)), 6.01-5.53 (broad, CH_2=C*H*- from the repeating units of PI), 5.20-4.32 (broad *m*, C*H*$_2$= from the repeating units of PI), 3.63 (2H, broad, -C*H*$_2$-OH), 2.32-0.50 (broad *m*, aliphatic protons).

According to NMR, number-average molecular mass (M_n) of OH-$PI_{4.3k}$-OH was estimated as 4.3 kg.mol^{-1}. This value will be used for the experimental calculations and the corresponding PI name OH-$PI_{4.3k}$-OH. The $M_{n,NMR}$ value was calculated by ^{1}H NMR chain-end analyses according to Equation 1:

$$M_{n,NMR}(OH - PI - OH) = M_{isoprene} * \left(\frac{2I_h}{I_a}\right) + M_{poly(\alpha-methylstyrene)} + M_{C_4H_{10}O_2} \qquad (1)$$

$$M_{isoprene} = 68\ \frac{g}{mol}$$

$M_{poly(\alpha-methylstyrene)} = 479\ \frac{g}{mol}$, initiator for the isoprene polymerization (data from the supplier)

$$M_{C_4H_{10}O_2} = 90\ \frac{g}{mol}$$

Synthesis of α,ω-diazido-terminated homoetelechelic PIs: The synthesis was adapted from procedures described in literature and adjusted for each individual PIs.[2,3] To exemplify, in a three-necked flask equipped with a magnetic stir bar, a solution of 6-azidohexanoic acid (877 mg, 5.58 mmol) in dry CH_2Cl_2 (10 mL) was added dropwise to a solution of OH-$PI_{4.3K}$-OH (3.0 g, 0.698mmol), DMAP (937 mg, 7.67 mmol) and EDC (1.47 g, 7.67 mmol) in dry CH_2Cl_2 (50 mL) under argon atmosphere. The reaction vessel was cooled with an ice bath during the addition and left stirring for 36 h at 34 C. N-hexane was added to the concentrated reaction mixture and washed three times with a saturated solution of K_2CO_3 and two times with water. The solution was dried with $MgSO_4$, filtrated and concentrated to give N_3-$PI_{4.3K}$-N_3 as a sticky white solid. *Yield 78%.*

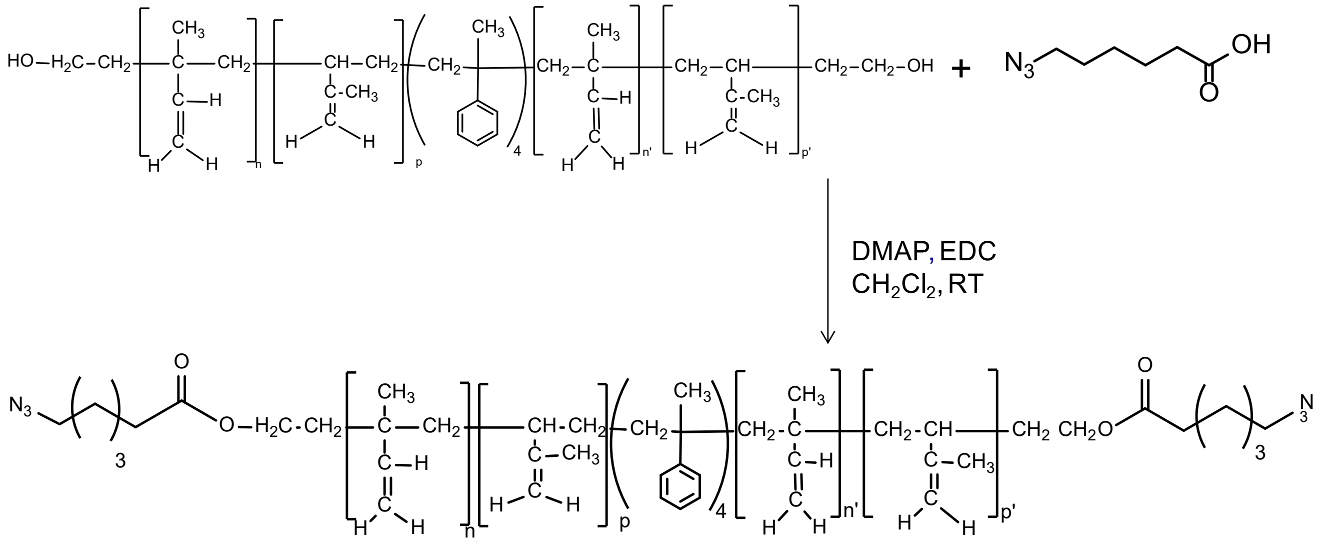

Figure S3. Synthesis of α,ω-diazido-terminated homotelechelic PIs (N_3-$PI_{4.3k/9.0k}$-N_3).

Peak assignments in the ^{1}H NMR spectrum of N_3-$PI_{4.3K}$-N_3 recorded in $CDCl_3$: 7.30-6.80 (broad, C_6H_5- from the PI initiator based on poly(α-methylstyrene)), 6.05-5.53 (broad, CH_2=C*H*- from the repeating units of PI), 5.21-4.33 (broad *m*, C*H₂*= from the repeating units of PI), 4.04 (4H, broad, -CH_2-O-C(=O)- (two end-groups)), 3.28 (4H, *triplet*, -CH_2-N_3 (two end-groups), 2.30 (4H, *m*, -O(C=O)-CH_2- (two end-groups)), 2.16-0.50 (broad *m*, aliphatic protons).

The ^{1}H NMR spectrum of N_3-$PI_{4.3k}$-N_3 shows that the signal at δ= 3.65 ppm, due to the methylene protons adjacent to the terminal hydroxyl groups in OH-$PI_{4.3k}$-OH (**Fig S1**), completely disappeared after the acylation whereas signals at 4.04(***a***) and 3.27(***l***) ppm, respectively corresponding to the methylenes in α-position of the -O-C(=O)- groups and next to the azido end-groups, remain (**Fig S4**). This is in accordance with the quantitative formation of N_3-$PI_{4.3k}$-N_3.

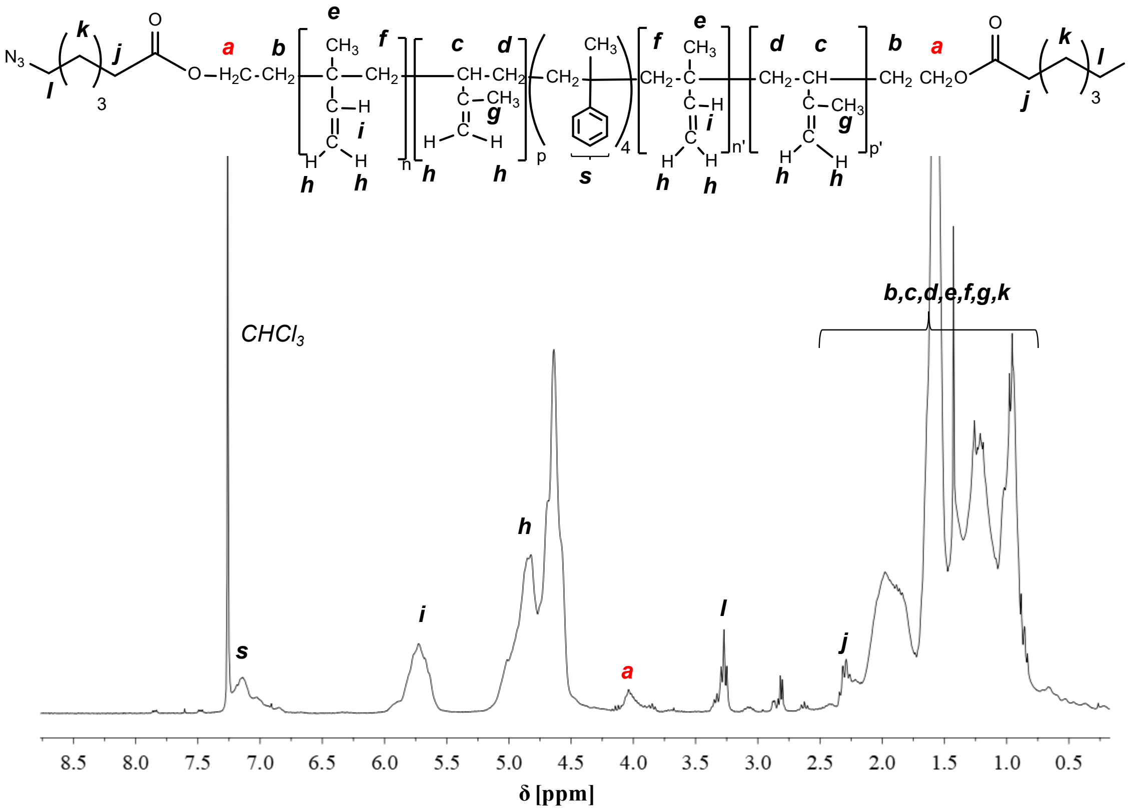

Figure S4. ^{1}H NMR spectrum (400 MHz; $CDCl_3$) of the α,ω-diazido-terminated homotelechelic PI N_3-$PI_{4.3k}$-N_3.

Syntheses of triBCPs $MH_{1.2k}$-(*T*)-$PI_{4.3k/9.0k}$-(*T*)-$MH_{1.2k}$: A solution of N_3-$PI_{4.3k}$-N_3 (1.50 g, 0.345 mmol) in tetrahydrofuran (THF) (13 mL) was degassed with high-purity argon for 10 min. The solution was then cannulated to a similarly degassed solution of MH-C≡CH (1.03 g, 0.837mmol) in dimethylformamide (DMF) (9 mL) and copper nanopowder (120.0 mg, 0.837 mmol) was added under an argon atmosphere. The mixture was stirred at 64°C until the infrared spectrum showed complete disappearance of the azido N_3-precursor, i.e. during 2 days. The reaction mixture was then filtered through celite and concentrated. To completely remove copper, the concentrate was redissolved in an excess of THF together with a few milliliters of water and charged with Cuprisorb resin. The mixture was stirred at 50 °C overnight, filtered through celite and concentrated by evaporation. The crude product was then purified by precipitation in cold methanol (MeOH). The copolymer was collected by filtration and dried a under dynamic primary vacuum (of ca. 10^{-2} mbar) to give $MH_{1.2k}$-$PI_{4.3k}$-$MH_{1.2k}$ as a white solid. The same procedure was utilized for N_3-$PI_{9.0k}$-N_3. *Yield 68%.*

Fourier-Transform Infra-Red (FT-IR) spectroscopy confirmed the successful completion of the click reaction with i) the disappearance of the signal at 2096 cm^{-1} assigned to the azido groups of N_3-$PI_{4.3K}$-N_3, and ii) the appearance of the hydroxyl band from *N*-maltoheptaosyl-3-acetamido-1-propyne at *ca.* 3300 cm^{-1} (**Fig S5**).

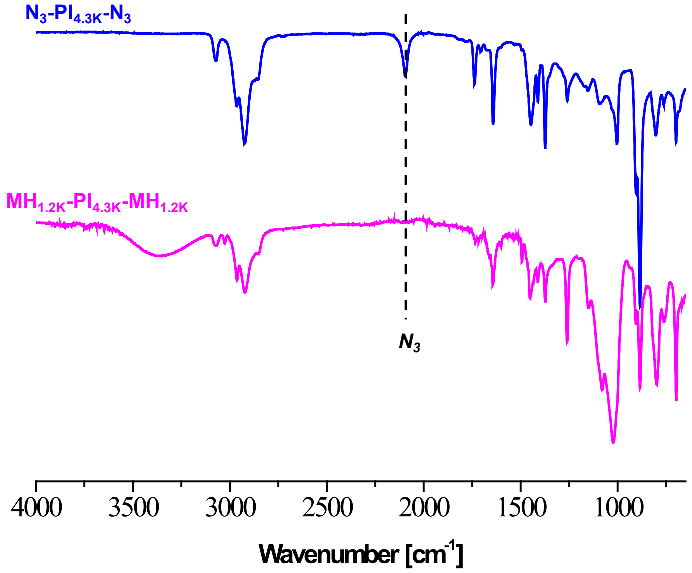

Figure S5. FT-IR spectra of N_3-$PI_{4.3k}$-N_3 and $MH_{1.2k}$-$PI_{4.3k}$-$MH_{1.2k}$.

^1H NMR spectrum of $MH_{1.2k}$-$PI_{4.3k}$-$MH_{1.2k}$ exhibits the presence of proton signals characteristics of both the PI and MH blocks together with the ones of the triazole ring (**Fig S6**).

Peak assignments in the ^1H NMR spectrum of $MH_{1.2k}$-(T)-$PI_{4.3k}$-(T)-$MH_{1.2k}$ recorded in THF-*d*$_8$ and additional 6 drops of DMSO-*d*$_6$: 7.85 (H from triazole), 7.30-6.80 (broad, C_6H_5- from the PI initiator poly(α-methylstyrene)), 6.05-5.62 (broad, CH_2=C*H*- from the repeating units of PI), 5.60-5.25 (broad *m*, OH from MH), 5.20-4.98 (broad *m*, H-1 from MH), 5.21-4.51 (broad *m*, C*H*$_2$= from the repeating units of PI), 4.50-4.40 (broad, OH from MH), 4.30 (*m*, -C*H*$_2$-N), 4.25-3.08 (broad *m*, H-2, 3, 4, 5, 6 from MH), 3.99 (broad, -C*H*$_2$-O-C(=O)-), 2.43-0.50 (broad *m*, aliphatic protons).

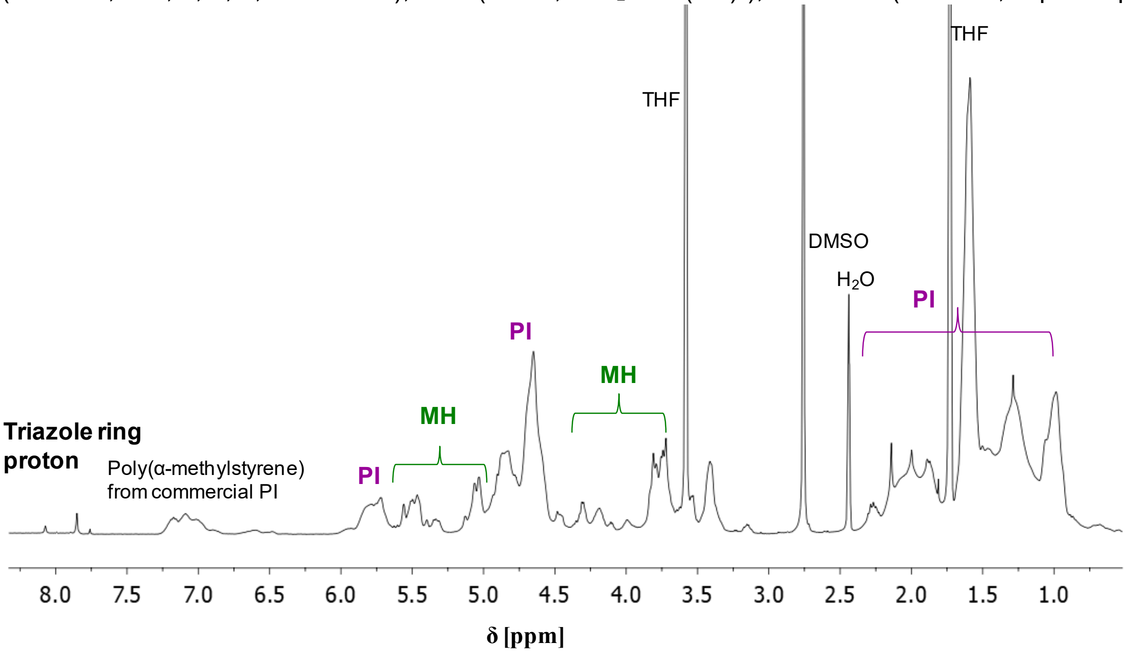

Figure S6. ^1H NMR spectrum of $MH_{1.2k}$-(T)-$PI_{4.3k}$-(T)-$MH_{1.2k}$. (400 MHz; THF-*d*$_8$ and 6 drops of DMSO-*d*$_6$)

Peak assignments in the ^{1}H NMR spectrum of $MH_{1.2k}$-(T)-$PI_{9.0k}$-(T)-$MH_{1.2k}$ recorded in THF-d_8 and additional 6 drops of DMSO-d_6: 7.8 (H from triazole), 7.30-6.50 (broad, C_6H_5- from the PI initiator poly(α-methylstyrene)), 6.05-5.6 (broad, CH_2=C*H*- from the repeating units of PI), 5.60-5.25 (broad *m*, OH from MH), 5.20-4.98 (broad *m*, H-1 from MH), 5.21-4.51 (broad *m*, CH_2= from the repeating units of PI), 4.50-4.40 (broad, OH from MH), 4.30 (*m*, -CH_2-N), 4.25-3.08 (broad *m*, H-2, 3, 4, 5, 6 from MH), 3.99 (broad, -CH_2-O-C(=O)-), 2.43-0.50 (broad *m*, aliphatic protons).

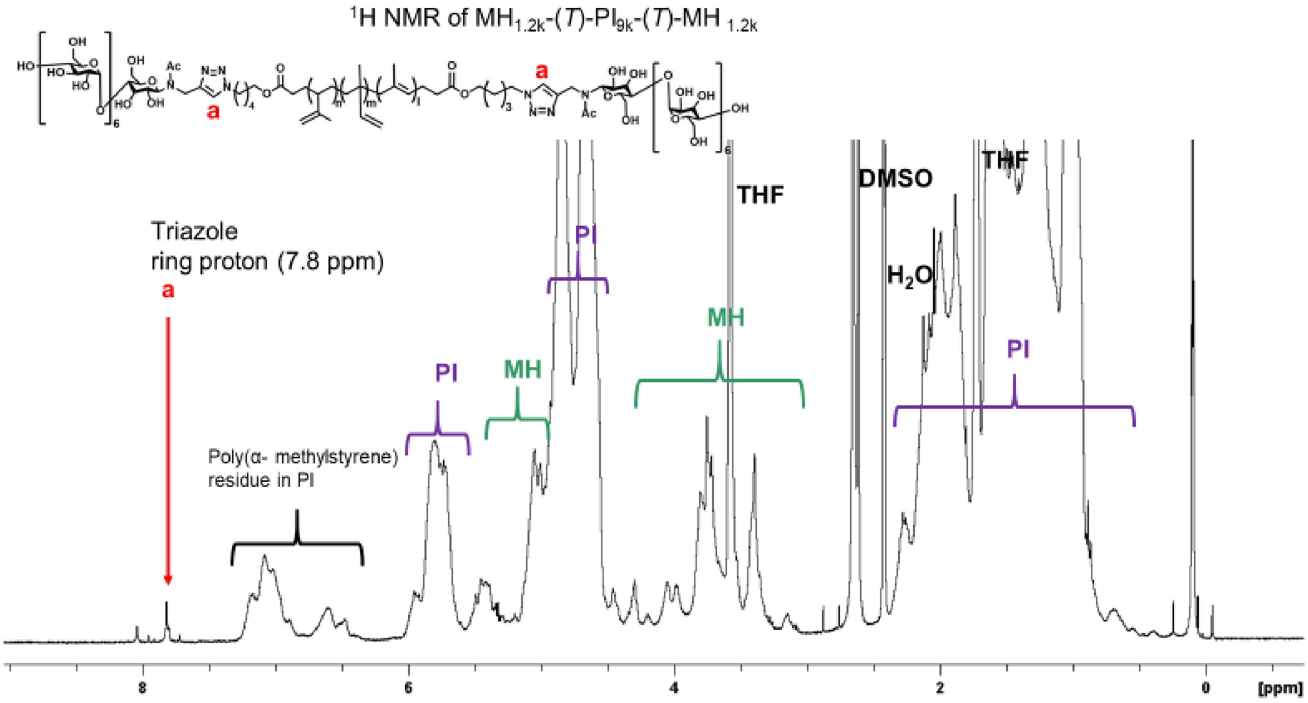

Figure S7. ^{1}H NMR spectrum of $MH_{1.2K}$-(T)-$PI_{9.0K}$-(T)-$MH_{1.2K}$. (400 MHz; THF-d_8 and 6 drops of DMSO-d_6)

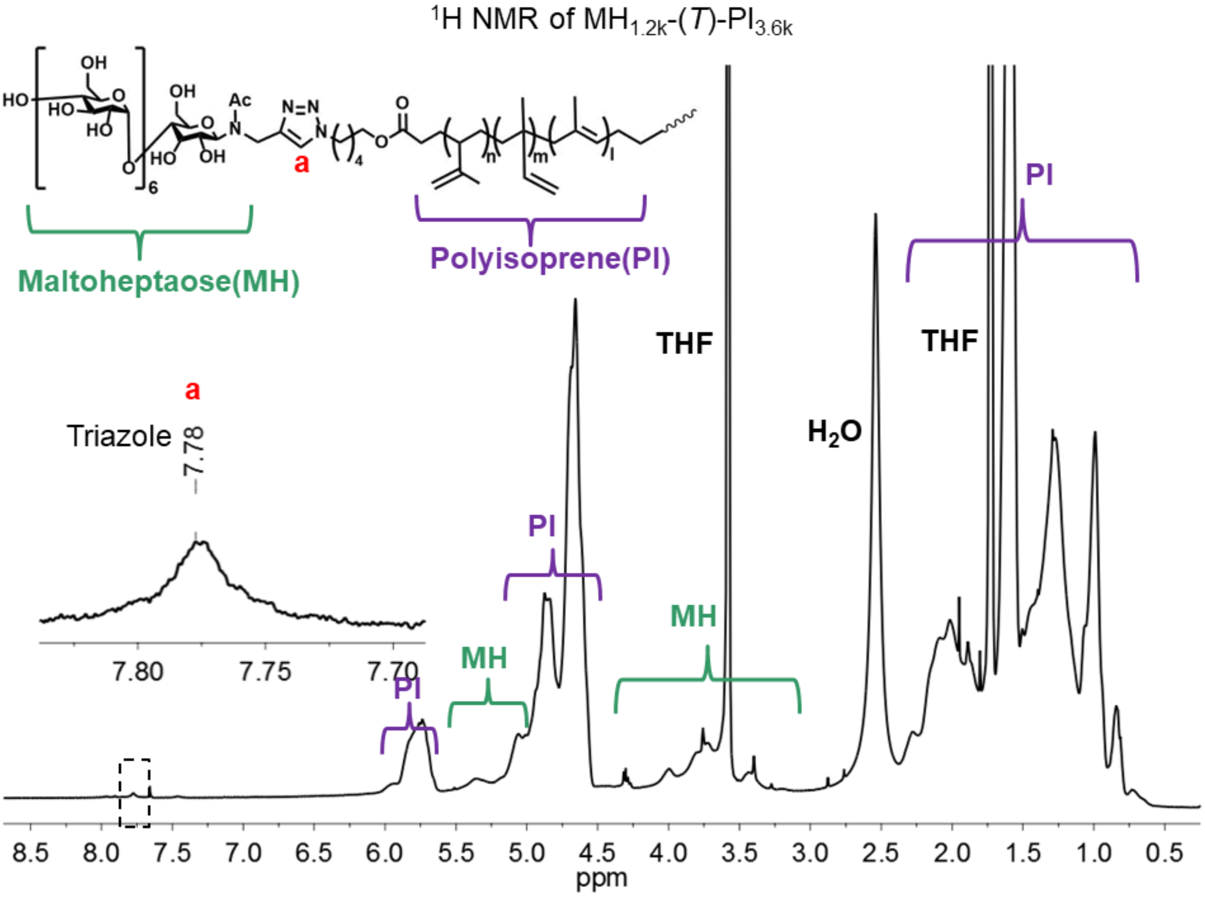

Figure S8. ^{1}H NMR spectrum of $MH_{1.2k}$-(T)-$PI_{3.6k}$ (400 MHz; THF-d_8).

***N*-alkylation of Triazole (T) rings with MeTFSI or MeI and subsequent characterizations**

General procedure: 100mg of diBCP $MH_{1.2k}$-(T)-$PI_{3.6k}$ or triBCP $MH_{1.2k}$-(T)-$PI_{4.3k}$-(T)-$MH_{1.2k}$ or $MH_{1.2k}$-(T)-PI_{9k}-(T)-$MH_{1.2k}$ was weighted to a round bottom flask and dissolved in 5 mL THF, with a gentle heating (at ca. 50°C). To obtain a clear solution, 0.5 mL of DMSO was added and vigorously stirred (magnetic stirring) at the end of the heating step. 20 molar equivalents of N-Methyl bis[(trifluoromethyl)sulfonyl]imide (MeTFSI) (or iodomethane (MeI)) with respect to the content of triazole rings within (Di or Tri-) BCP was added in 0.5 mL of THF to the reaction medium. Magnetic stirring at 60°C was conducted during 16 hours. The *n*-alkylation reaction was quenched by cooling down to Room Temperature (i.e. RT=25°C) **(Fig S9)** THF and extra *n*-alkylation reagent (MeTFSI or MeI) were removed by rotary evaporation. 2 ml of THF was then added and the product dialyzed against Milli-Q® water *(i.e. resistivity of ca. 18.2 MΩ.cm^{-1} at 25 °C)* for a period of one week. Dialyses water was changed three times the first day (d1) and one time per day during the d2-d7 period. The final product was lyophilized from water. All products were characterized by ^{1}H NMR spectroscopy. (Fig **S10**)

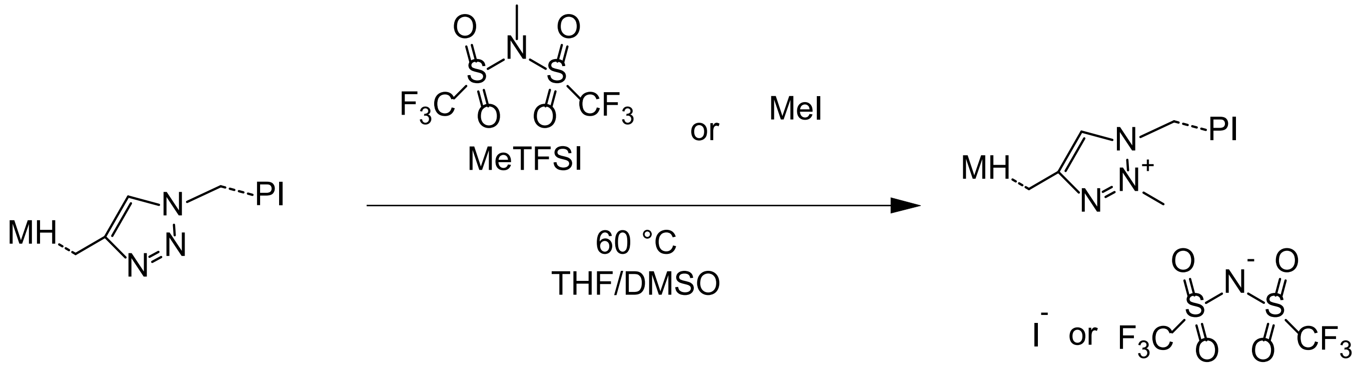

Figure S9. Reaction scheme for the *n*-alkylation of triazole ring with MeTFSI or MeI toward a charged methyltriazolium$^+$/TFSI$^-$ (or methyltriazolium$^+$/I$^-$) interface taking MH-(T)-PI diBCP as a representative example. Note that the same *n*-alkylation procedure was applied to the MH-(T)-PI-(T)-MH triBCPs.

a) ^1H NMR of $MH_{1.2k}$-($T^+/TFSI^-$)-$PI_{3.6k}$

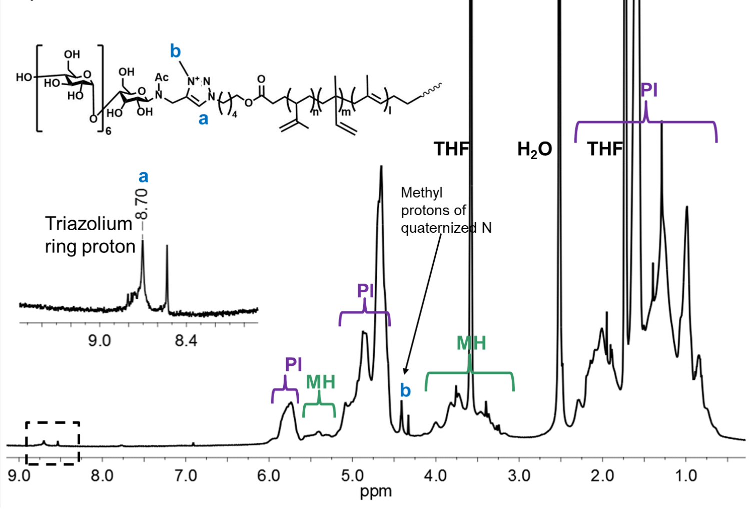

b) ^1H NMR of $MH_{1.2k}$-($T^+/TFSI^-$)-$PI_{4.3k}$-($T^+/TFSI^-$)-$MH_{1.2k}$

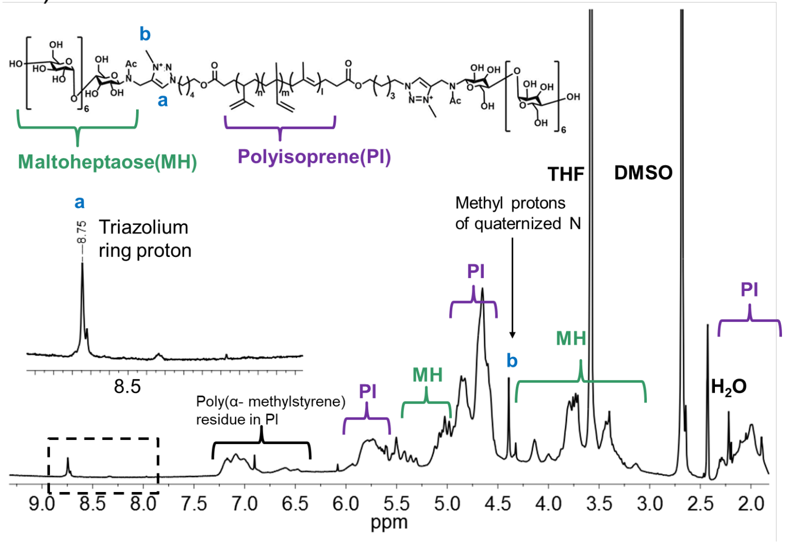

c) ^1H NMR of $MH_{1.2k}$-($T^+/TFSI^-$)-PI_{9k}-($T^+/TFSI^-$)-$MH_{1.2k}$

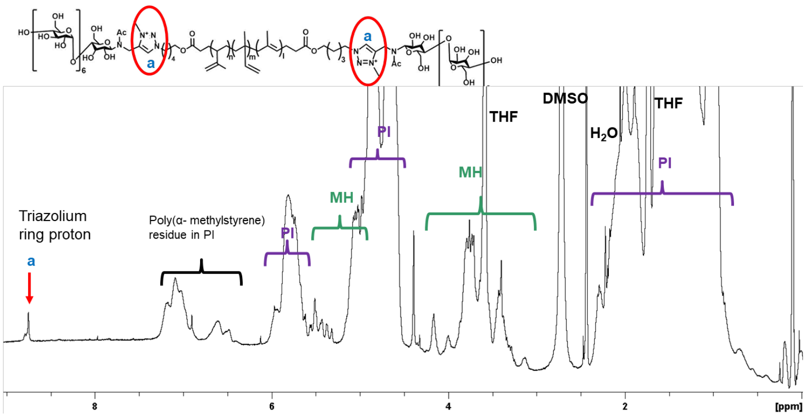

Importantly and contrary to the case in which MeTFSI with used as the *n*-alkylation reagent, we noted that the *n*-alkylation of di/triBCPs using MeI never ended into full conversion. Indeed, unreacted triazole rings are clearly maintained in $MH_{1.2k}$-(T^+/I^-)-$PI_{3.6k}$ and $MH_{1.2k}$-(T^+/I^-)-$PI_{4.3k}$-(T^+/I^-)-$MH_{1.2k}$ and $MH_{1.2k}$-(T^+/I^-)-PI_{9k}-(T^+/I^-)-$MH_{1.2k}$ as shown by ^1H-NMR spectroscopy, even if reaction times were prolonged up to 16 days or n-alkylation reagent (MeI) amount increased. The *triazole (T)-to-methyltriazolium (T^+)* conversions were 60, 50 and 11 % respectively, calculated from the corresponding ^1H NMR integrals. (see **S11**)

a) ^{1}H NMR of $MH_{1.2k}$-(T^{+}/I^{-})-$PI_{3.6k}$

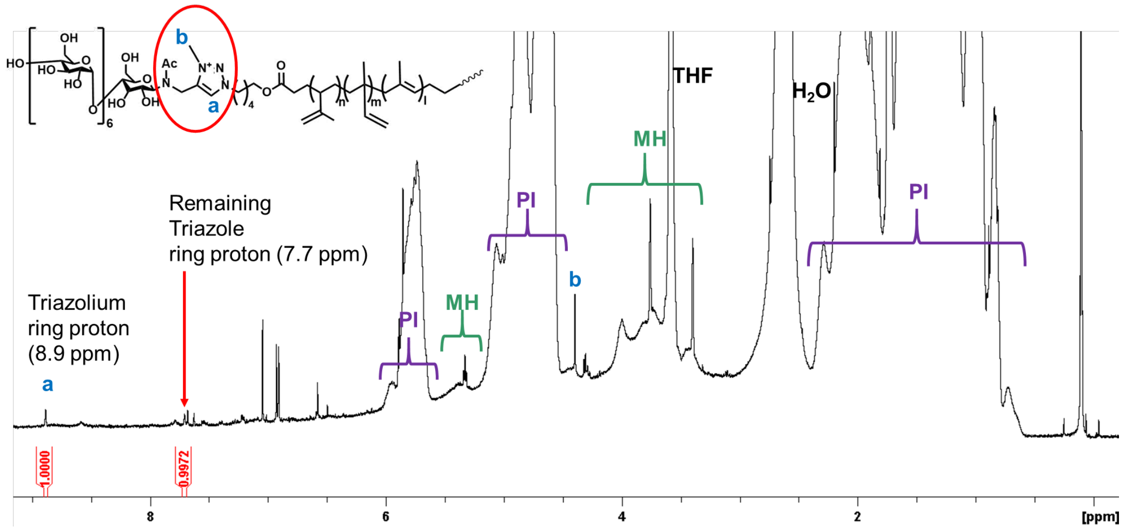

b) ^{1}H NMR of $MH_{1.2k}$-(T^{+}/I^{-})-$PI_{4.3k}$-(T^{+}/I^{-})-$MH_{1.2k}$

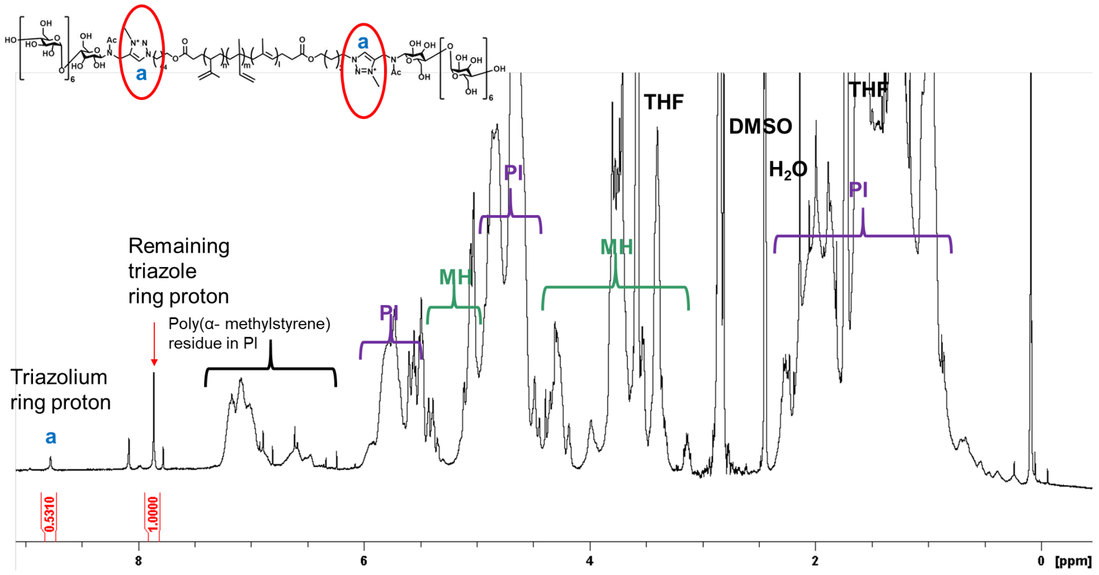

c) ^{1}H NMR of $MH_{1.2k}$-(T^{+}/I^{-})-PI_{9k}-(T^{+}/I^{-})-$MH_{1.2k}$

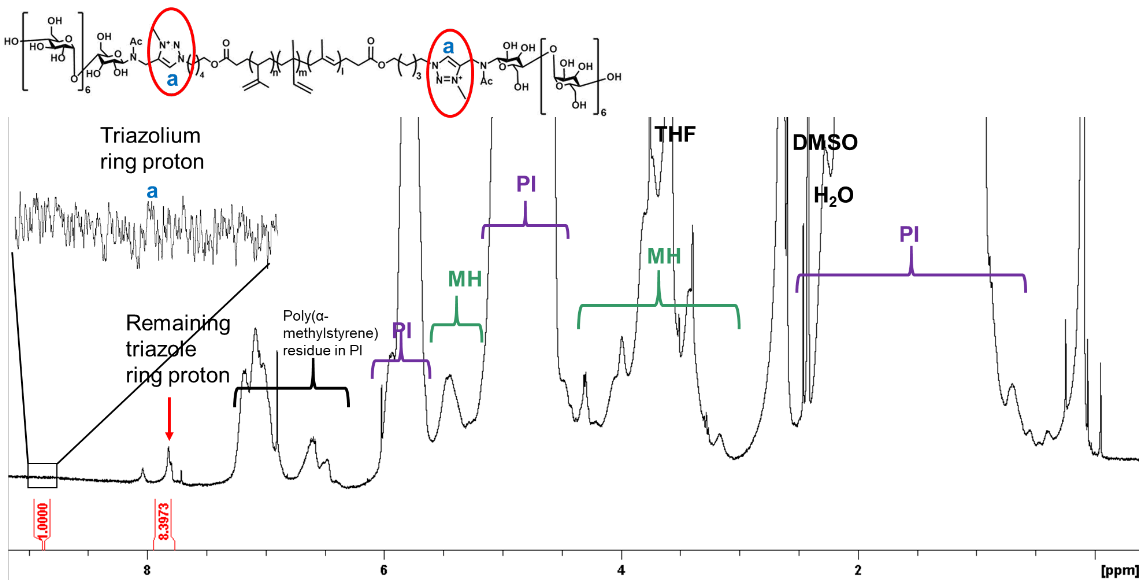

Differential Scanning Calorimetry (DSC)

DSC analyses were performed using a TA Instruments DSC Q200 equipped with a RCS 90 cooling unit. Materials (weight of ca. 5 mg) were analyzed using aluminum sample pans. Measurements were carried out at a scan rate of 5 °C.min^{-1} under a nitrogen atmosphere. All BCPs showed a glass transition temperature (determined during a second cooling scan performed at a scan rate of 20°C.min^{-1}) spanning over the of 5-19 ^{o}C temperature range.

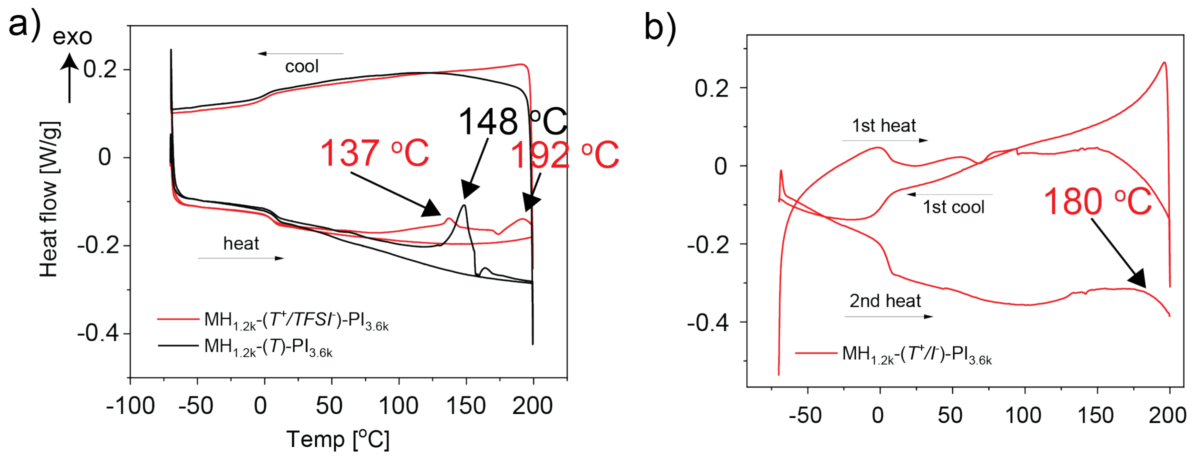

Figure S12. DCS heating/cooling/heating cycles for a) **pristine** vs. **MeTFSI**-modified/charged **diBCP** and b) for **MeI**-modified/charged **diBCP**. In a), the second heating scan onto MeTFSI-modified/charged diBCP did not reproduce the exothermic peaks seen upon the first heating scan. MeTFSI-modified/charged diBCP does not show any peaks during the heating cycles.

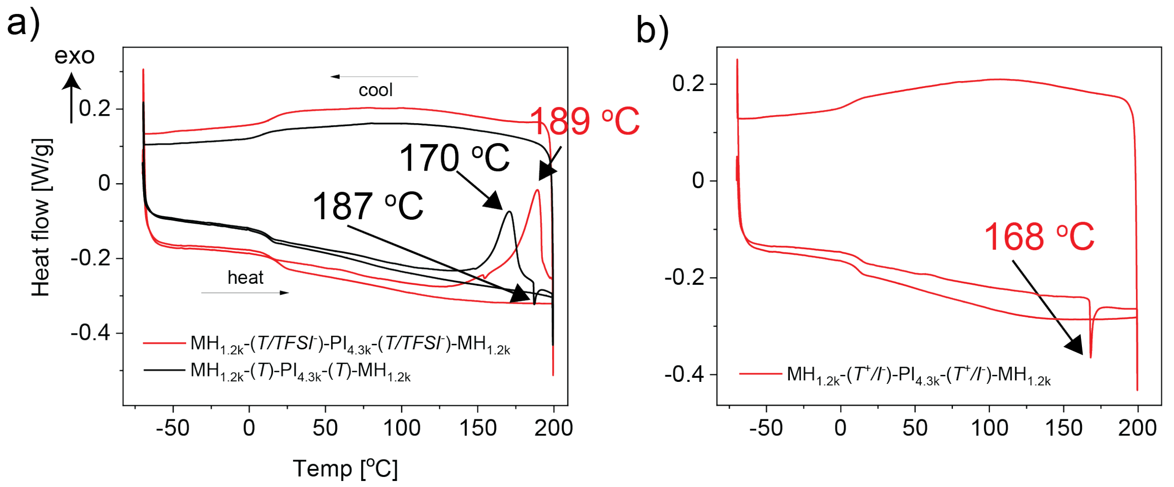

Figure S13. DCS heating/cooling/heating cycles for a) pristine and **MeTFSI**-modified/charged **triBCP (M_n PI= 4300 g.mol^{-1})** and **b)** for **MeI**-modified/charged **triBCP**. In all cases, the second heating did not reproduce the peaks seen upon 1st heating.

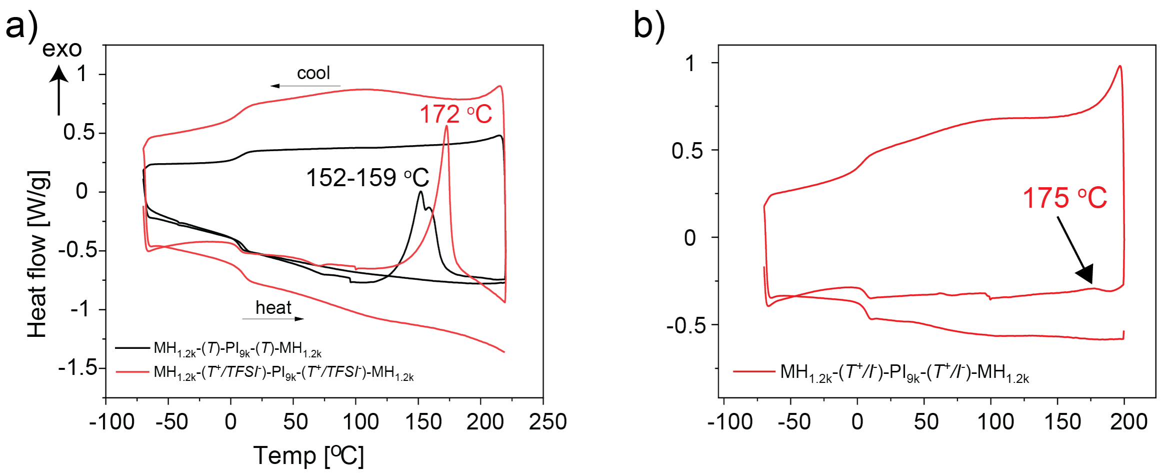

Figure S14. DCS heating/cooling/heating cycles for a) pristine and **MeTFSI**-modified/charged **triBCP (M_n PI= 9000 g.mol^{-1})** and **b)** for **MeI**-modified/charged **triBCP**. In all cases, the second heating did not reproduce the peaks seen upon 1st heating.

Variable temperature Small Angle X-ray Scattering (SAXS)

SAXS experiments were carried out on the BM02-D2AM French (CRG) beamline at the European Synchrotron Radiation Facility (ESRF, Grenoble, France). The BCP samples were placed into glass capillaries with an inner diameter of 1.5 mm. SAXS profiles were obtained by 5 °C steps during a continuous heating process from 25 to 185 °C (heating rate, ca. 2.5 °C min^{-1}; λ = 0.689 Å; acquisition time, 20 sec).

Thin film preparation

General procedure: 5 mg/mL BCP solution ($MH_{1.2k}$-(T^+/$TFSI^-$)-$PI_{4.3k}$-(T^+/$TFSI^-$)-$MH_{1.2k}$ in THF and pristine triBCP $MH_{1.2k}$-(T)-$PI_{4.3k}$-(T)-$MH_{1.2k}$ in THF/DMSO 90/10 wt % mixture) were spin-coated (3000rpm, 60s) on plasma-treated Si wafer. Next, films were Solvent Vapor Annealed (SVA) with THF/H2O 90/10 wt % solvent mixture for 24h. Swollen thin films were dried overnight at RT (ca. 25°C) before further characterizations by AFM. Film thicknesses of *ca.* 21 nm were measured by AFM.

Atomic Force Microscopy (AFM): Additional images

AFM images were taken with aPicoPlus (Molecular Imaging, Corp., Tempe, AZ) microscope in the tapping mode with silicon probes (Mikromasch HQ: NSH16/A1 BS) or with a Dimension Icon (Bruker, Billerica, MA, USA) microscope with SCAN ASIST-Air probes in the peak-force mode.

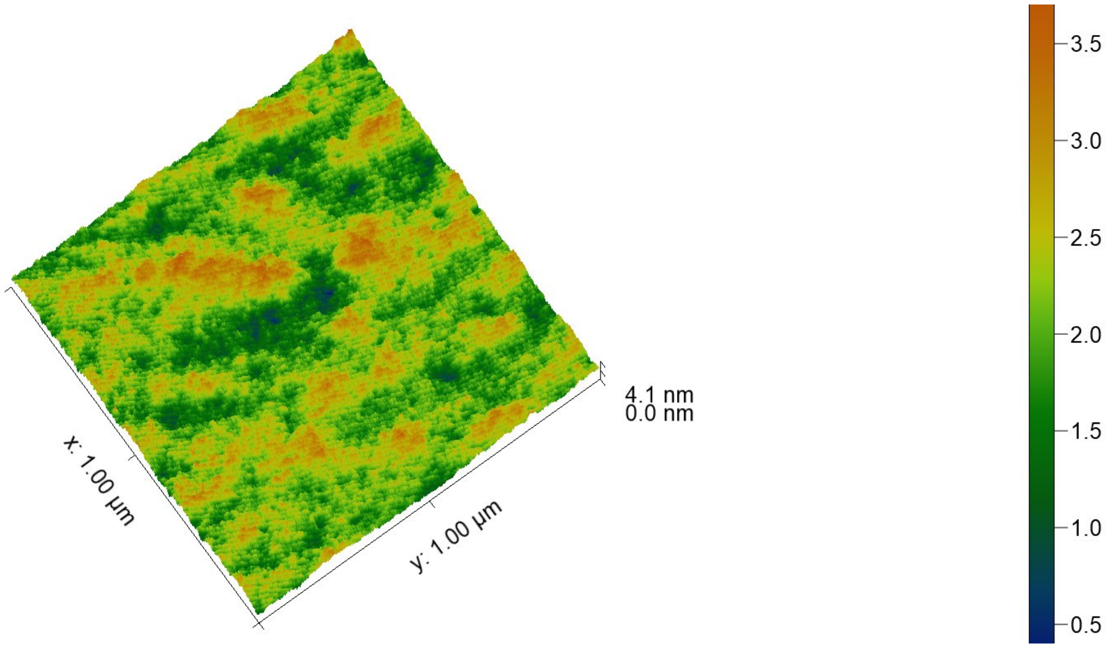

Figure S15. AFM 3D height image of $MH_{1.2k}$-(T^+/$TFSI^-$)-$PI_{4.3k}$-(T^+/$TFSI^-$)-$MH_{1.2k}$ thin film demonstrating average ~4 nm height differences on the top layer. Collapsed polyisoprene layer protruding at the air-polymer interface amounts ca.1.5 nm in height. The overall line features remain visible and crossing the 1x1 µm image unidirectionally due to ionic interactions developing at the charge interface between alternating maltoheptaose (MH) and polyisoprene (PI) lamella layers perpendicular to the substrate surface.

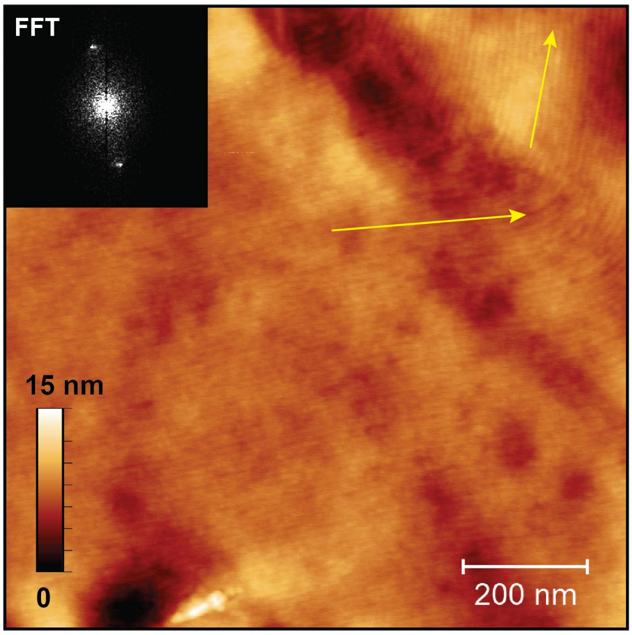
FFT
15 nm
0
200 nm

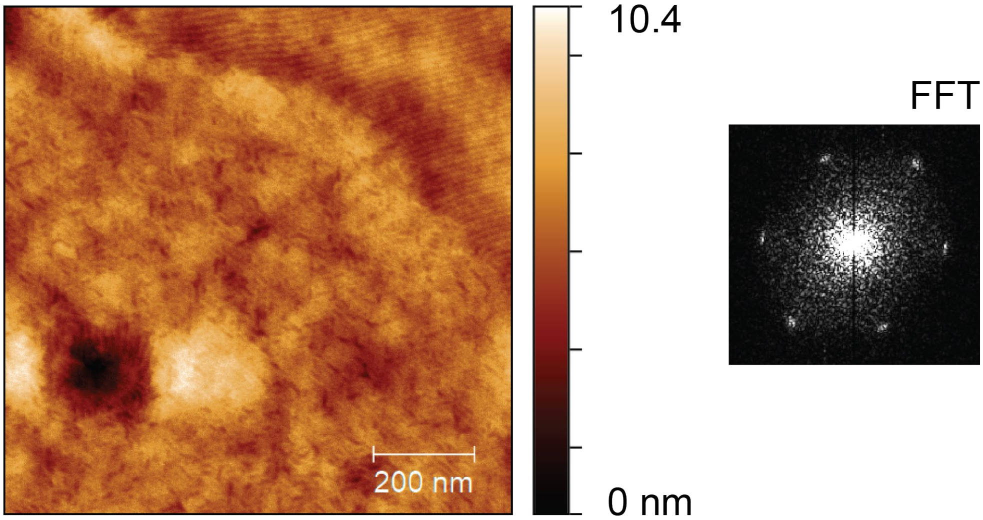
200 nm
10.4
0 nm
FFT

Grazing Incidence Small Angle X-ray Scattering (GISAXS)

GISAXS measurements were performed at the D2AM beamline (ESRF, Grenoble France). 16 keV energy was used with a 0.11° incident beam angle. 10-100s scans were taken. First sample was rotated by ±30° for signal optimization, following 100s data collection for the chosen angle. The raw data was analyzed with fitGISAXS[6] using IGOR Pro and FIT2D programs.

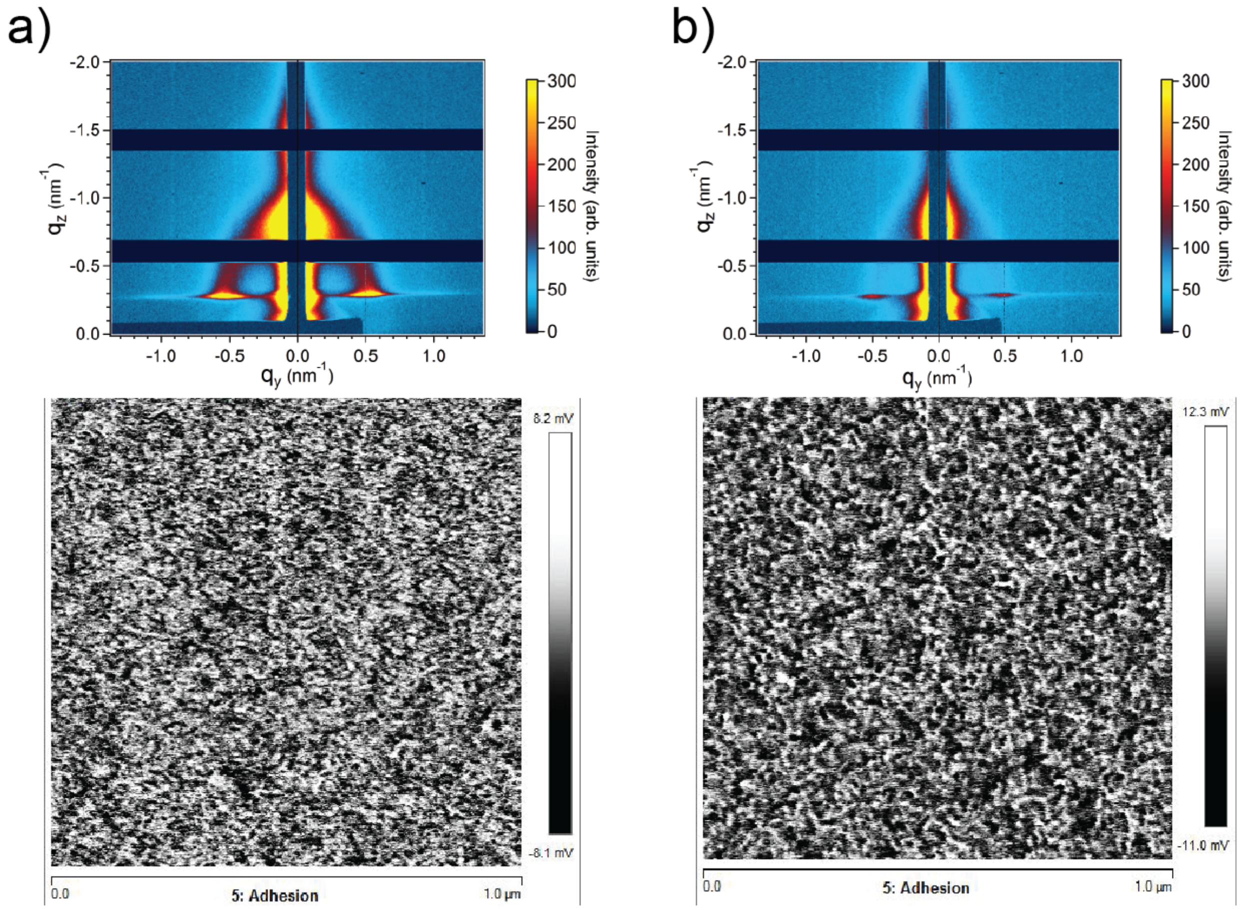

Figure S18. 2D GISAXS and AFM images for as-cast **a)** $MH_{1.2k}$-(T^+/$TFSI^-$)-$PI_{4.3k}$-(T^+/$TFSI^-$)-$MH_{1.2k}$ and **b)** $MH_{1.2k}$-(T)-$PI_{4.3k}$-(T)-$MH_{1.2k}$ triBCP thin films.

Supplementary References

(1) Otsuka, I.; Fuchise, K.; Halila, S.; Fort, S.; Aissou, K.; Pignot-Paintrand, I.; Chen, Y.; Narumi, A.; Kakuchi, T.; Borsali, R. Thermoresponsive Vesicular Morphologies Obtained by Self-Assemblies of Hybrid Oligosaccharide-Block-Poly(N-Isopropylacrylamide) Copolymer Systems. *Langmuir* **2010**, *26*, 2325–2332.

(2) Isono, T.; Otsuka, I.; Suemasa, D.; Rochas, C.; Satoh, T.; Borsali, R.; Kakuchi, T. Synthesis, Self-Assembly, and Thermal Caramelization of Maltoheptaose-Conjugated Polycaprolactones Leading to Spherical, Cylindrical, and Lamellar Morphologies. *Macromolecules* **2013**, *46*, 8932–8940.

(3) Grandjean, C.; Boutonnier, A.; Guerreiro, C.; Fournier, J. M.; Mulard, L. A. On the Preparation of Carbohydrate-Protein Conjugates Using the Traceless Staudinger Ligation. *J. Org. Chem.* **2005**, *70*, 7123–7132.

(4) Hung, C. C.; Chiu, Y. C.; Wu, H. C.; Lu, C.; Bouilhac, C.; Otsuka, I.; Halila, S.; Borsali, R.; Tung, S. H.; Chen, W. C. Conception of Stretchable Resistive Memory Devices Based on Nanostructure-Controlled Carbohydrate-Block-Polyisoprene Block Copolymers. *Adv. Funct. Mater.* **2017**, *27*, 1606161.

(5) Konrad, M.; Knoll, A.; Krausch, G.; Magerle, R. Volume Imaging of an Ultrathin SBS Triblock Copolymer Film. *Macromolecules* **2000**, *33*, 5518–5523.

(6) Babonneau, D. FitGISAXS: Software Package for Modelling and Analysis of GISAXS Data Using IGOR Pro. *J. Appl. Crystallogr.* **2010**, *43*, 929–936.
